# Direct pharmacological AMPK activation inhibits mucosal SARS-CoV-2 infection by reducing lipid metabolism, restoring autophagy flux and the type I IFN response

**DOI:** 10.1128/jvi.00394-25

**Published:** 2025-06-12

**Authors:** Andrea Cottignies-Calamarte, Flora Marteau, Feifan He, Sandrine Belouzard, Jean Dubuisson, Daniela Tudor, Benoit Viollet, Morgane Bomsel

**Affiliations:** 1 Laboratory of Mucosal Entry of HIV and Mucosal Immunity Institut Cochin, Paris, France; 2 Université Paris Cité, CNRS, Inserm, Institut Cochin 555089, Paris, France; 3 Virologie moléculaire et cellulaire des coronavirus, Center d’infection et d’immunité de Lille, Institut Pasteur de Lille, Université de Lille, CNRS, Inserm, CHRU 27023, Lille, France; St. Jude Children's Research Hospital, Memphis, Tennessee, USA

**Keywords:** SARS-CoV-2, AMP-activated protein kinase, autophagy, lipid metabolism, antiviral agents

## Abstract

AMP-activated protein kinase (AMPK) plays a central role in regulating cell energy balance. When activated, AMPK suppresses energy-consuming pathways, such as lipid and protein synthesis, while increasing nutrient availability through the activation of autophagy. These pathways downstream of AMPK activation contribute to SARS-CoV-2 infection, which hijacks autophagy and accumulates lipid droplets in viral factories to support viral replication. Here, we assessed the antiviral activity of the direct pan-AMPK allosteric activator MK-8722 *in vitro*. MK-8722 efficiently inhibited infection of Alpha and Omicron SARS-CoV-2 variants in Vero76 and human bronchial epithelial Calu-3 cells at micromolar concentration. This inhibition relied on restoring the autophagic flux, which redirected newly synthesized viral proteins for degradation, and reduced lipid metabolism, which affected viral factories. Furthermore, MK-8722 treatment increased the type I interferon (IFN-I) response. Post-infection treatment with MK-8722 was enough to inhibit efficient viral replication and restore the IFN-I response. Finally, MK-8722 treatment did not alter the SARS-CoV-2-specific CD8^+^ T cell response mounted upon Spike vaccination. Overall, by activating AMPK, MK-8722 acts as an effective antiviral against SARS-CoV-2 infection, even when applied post-exposure, paving the way for preclinical tests aimed at inhibiting viral replication and improving patients’ symptoms.

**IMPORTANCE:**

Coronavirus disease 2019, caused by SARS-CoV-2 infection, has led to severe acute respiratory syndrome with very high mortality. Despite available vaccines and public health measures, new SARS-CoV-2 variants emerge with increased transmissibility requiring the development of novel therapeutic strategies. Recently, the AMP-activated protein kinase (AMPK), a cellular energy sensor, has emerged as a potential broad-spectrum antiviral target, as AMPK can modulate the intracellular environment in turn impeding viral replication. This study aims to evaluate the potential of pharmacological activation of AMPK to inhibit SARS-CoV-2 infection and replication. Our findings demonstrate that AMPK activation induces significant alterations in host cellular lipid metabolism that disrupt viral factories essential for SARS-CoV-2 replication. Furthermore, by enhancing autophagy, a process crucial for the degradation and clearance of viral particles, AMPK activation facilitates the elimination of the virus. Therefore, targeting AMPK signaling pathways could offer a promising therapeutic approach for the treatment of SARS-CoV-2 infections.

## INTRODUCTION

The coronavirus disease 2019 (COVID-19) pandemic was efficiently contained due to the rapid development of vaccines and efficient public health policies. However, despite these efforts, SARS-CoV-2 variants of concern, which are poorly sensitive to these vaccines, emerged and spread in the population, resulting in millions of deaths (1). Meanwhile, it has been challenging to develop an efficient antiviral treatment with an acceptable benefit/risk ratio. As of January 2025, the treatment of patients in the intensive care unit remains primarily limited to drugs that either inhibit viral components such as Remdesivir and Paxlovid or target SARS-CoV-2-induced cytokine storm, such as the IL-6 receptor antagonist Tocilizumab, and developing antiviral remains a timely task (1–3).

After its nasal entry, SARS-CoV-2 primarily infects lung epithelial cells in a mechanism by which the viral envelope spike protein (Spike) binds to its main receptors angiotensin-converting enzyme 2 (ACE2) and/or transmembrane protease serine 2 (TMPRSS2). Following viral entry and the delivery of viral RNA into the cytosol, the viral genes ORF1a and ORF1b are translated to RNA-dependent RNA-polymerase (RdRp). The viral RdRp further generates the remaining viral mRNA, and the viral cycle subsequently proceeds by hijacking cellular processes (2). Replication of SARS-CoV-2, similar to other enveloped RNA viruses, is supported by so-called viral factories with highly curved and three-dimensional folded membrane extensions of modified endoplasmic reticulum (4, 5), whose formation requires an increase in cell lipid metabolism. Viral factories compartmentalize viral genome from the cytosol for replication and transcription and provide protection against host cell defense. Disrupting lipid metabolism hinders viral replication (6–8). Accordingly, SARS-CoV-2 infection results in a significant alteration in the cellular lipid repertoire in favor of polyunsaturated fatty acids, which are necessary for the generation of infectious viruses (9–12). This shift in lipid profile is mainly driven by the viral proteins ORF9, nsp4, and nsp6, leading to the accumulation of lipid droplets (LDs) in infected cells *in vitro* and in COVID-19 autopsy tissues (6, 13–16). Accordingly, disrupting chemically either of these mechanisms inhibits SARS-CoV-2 replication (6, 12, 13, 15, 17).

Throughout the SARS-CoV-2 viral cycle, autophagy is sequentially activated and inhibited (18–21). Indeed, autophagy is initiated by the early expressed nsp6, resulting in the formation of autophagosomes that are essential for the establishment of viral factories (18, 22) and subsequent viral protein expression (18, 22). In turn, viral proteins OFR3a and ORF7a expressed at later time post-infection prevent the fusion between autophagosomes and lysosomes, thereby blocking the completion of autophagy, as evidenced by increased LC3-B expression and accumulation of the autophagy cargo receptor sequestosome-1/p62 (18, 22). Overall, this disruption of autophagy protects the newly formed virus from degradation in the LAMP1^+^ lysosome (20, 23–26).

SARS-CoV-2 infection also impairs the expression of interferon type 1 (IFN-I) and interferon-stimulated genes (ISG), such as *Mx1* or *OAS1*. This reduction fails to restrict SARS-CoV-2 replication, and this contributes to the hyperinflammatory symptoms observed in COVID-19 (27–30). Finally, SARS-CoV-2 infection interferes with the establishment of an efficient cellular response. By escaping lysosomal degradation, SARS-CoV-2 limits viral peptide loading onto MHC-I and subsequent presentation to specific cytotoxic CD8^+^ T lymphocytes (CTL) (31, 32). In addition, SARS-CoV-2 ORF8 down-regulates MHC-I expression from the plasma membrane, making infected cells less sensitive to SARS-CoV-2-specific CTLs (33).

AMP-activated protein kinase (AMPK) is a highly conserved cellular energy sensor that plays a crucial role in restoring the cellular energy balance by inhibiting ATP-consuming pathways (anabolism) and activating ATP-producing pathways (catabolism) (34). Notably, AMPK is a central regulator of lipid and protein metabolism. AMPK inhibits fatty acid and cholesterol synthesis by direct phosphorylation and inhibition of acetyl-CoA carboxylase (ACC) and 3-hydroxy-3-methylglutaryl-CoA (HMG-CoA) reductase, respectively (34). Additionally, AMPK is vital for the regulation of protein synthesis and cell growth through the inactivation of mTORC1 signaling through phosphorylation of tuberous sclerosis complex 2 and Raptor at position S792 (34). Moreover, by activating Unc-51-like autophagy-activating kinase 1 (ULK1) after phosphorylating S555 and stimulating nuclear factor erythroid 2-related factor 2 (NRF2)-dependent p62 increased expression, AMPK activation finely tunes cellular metabolic pathways (34). AMPK appears as a critical hub of cellular pathways affected by SARS-CoV-2 that are crucial for viral replication and pathogenesis.

A multitude of indirect and direct AMPK activators have been tested in preclinical models in various domains including viral infection and metabolic diseases (34–36). Recently, MK-8722, a direct allosteric activator of AMPK, has been developed (37). MK-8722 is effective *in vitro* in the micromolar range on AMPK activation, compared to the millimolar range for metformin and 5-aminoimidazole-4-carboxamide riboside (AICAR) (37, 38). MK-8722 has already been orally tested at 10 mg/kg in preclinical models of diabetes, resulting in improvements in glycemia but with minor and reversible cardiac hypertrophy (37).

Here, we investigated whether allosteric AMPK activation by MK-8722 inhibits SARS-CoV-2 infection/replication by promoting the autophagy of viral particles and restoring normal lipid metabolism and the IFN-I response.

## MATERIALS AND METHODS

### Cell culture and chemical compounds

All cells were cultured at 37°C in a humid atmosphere with 5% CO_2_. Vero76 (ATCC CRL-1587), Calu-3 (ATCC HTB-55), and Caco2 (ATCC HTB-37) cells were cultured in DMEM (Gibco 41966–029) containing 1% penicillin-streptomycin (Gibco 15140122) and decomplemented fetal calf serum (FCS; Eurobio CVFSVF06-01) at 10% (D10) for Calu-3 and Vero76 cells and at 20% (D20) for Caco2 cells. Caco2 cells culture medium was supplemented with 1% MEM Non-Essential Amino Acids (Gibco 11140035). All viral inoculations and infections were made in DMEM containing 2% FCS (D2).

Peripheral blood mononucleated cells (PBMCs) of healthy donors were collected from the French blood bank (Etablissement Français du Sang) after the initiation of SARS-CoV-2 vaccination in the global population (June 2021). PBMCs were obtained by Ficoll gradient centrifugation, aliquoted, and stored in StemMACS CryoBrew (Miltenyi, 130–109-558) until use. CD8^+^ T cells were isolated from HLA-A2^+^ individuals using CD8 microbeads (Miltenyi 130–045-201) following manufacturer instructions. Cells (5 × 10^6^ cells at 10^6^ cells/mL) were further expanded with TransAct (Miltenyi, 130–111-160, 1:100 dilution) for 15 days in RPMI 10% FCS and 2 mM L-glutamine (R10; Thermo 25030081) +10 IU/mL IL-2 (Roche 11011456001) after CD3/CD28 stimulation. TransAct was then washed out, and cells are frozen (1 million cells/vial) as described above. CD14-depleted PBMCs from healthy donors were obtained by collecting the flow-through of CD14 magnetic isolation (Miltenyi, 130–050-201). The resulting cells were then frozen as described above.

The absence of mycoplasma in the cell lines was routinely controlled using the MycoAlert mycoplasma detection kit (Lonza, LT07-318).

Remdesivir and MK-8722 were purchased at MedChemExpress (HY-104077 and HY-111363, respectively) and solubilized in dimethyl sulfoxide (DMSO) at 10 µg/mL.

### Virus propagation

SARS-CoV-2 variant Alpha and Omicron were obtained as we described (39, 40). Viral stocks were prepared by amplification in Vero76 cells (35), aliquoted, and cryopreserved at −80°C. Viral stocks were titrated in Vero76 cells after thawing, as previously described (35).

### Foci forming assay

Hundred thousand Vero76 cells were seeded in 48-well plates 1 day before infection, resulting in confluent cells on the day of infection. Virus-containing supernatant from infected cells to be tested were diluted at least 1,000 times in 100 µL of D2 and added to cells for 2 h and then replaced with an overlay medium (300 µL) composed of 0.3% low-melting agarose (Sigma A0701) in D2 at 37°C. Plates were then placed at 4°C for 10 min to allow the overlay medium to solidify and further incubated for 18 h at 37°C. Plates were washed three times with phosphate buffered saline (PBS) (Gibco 25300) to remove overlay, fixed with 4% paraformaldehyde (PFA) (Electron Microscopy Sciences 15710) in PBS (PBS-4% PFA) for 1 h at room temperature, and permeabilized with 0.1% Triton-X100 in PBS for 10 min. Cells were washed three times, and the viral nucleocapsid was labeled using a biotinylated-anti-N-antibody at 300 ng/mL (Bioss bsm-41411M-biotin) in PBS supplemented with 0.1% Saponin (Sigma S-7900), 2% FCS, and 2 mM EDTA (Perm Buffer) for 30 min at room temperature. Cells were washed three times in PBS, incubated with streptavidin-coupled-HRP 0.1 µg/mL (Vector Laboratories, INC, Burlingame, CA, 94010) in Perm Buffer for 30 min, washed again, and incubated with 100 µL/well of KPL TMB TrueBlue (Sera Care 5510–0030) for 30 min at 37°C.

The infectious index was established as the ratio of the viral copy number per milliliter over the focus forming unit (FFU) per milliliter titer computed from paired data sets obtained in reverse transcriptase quantitative PCR (RT-qPCR) and titration experiments.

### Inhibition assay

Vero76 and Calu-3 cells were plated, respectively, at least 1 and 5 days before infection. Cells were treated with MK-8722 either continuously or only after infection (post-infection). Continuous treatment consisted of treatment at indicated concentrations for 1 h before infection at 37°C, during the 2 h inoculation, and until endpoint. Fully confluent cell layers were infected with a multiplicity of infection (MOI) of 0.001 for Vero76 cells or of 0.05 for Calu-3 in D2 for 2 h. Inoculates were replaced by fresh D2 containing or not MK-8722, and cells were further incubated for 1 and 4 days for Vero76 and Calu-3 cells, respectively. Before harvesting cells, supernatants were collected, clarified by centrifugation, and stored at −80°C for further RT-qPCR quantification and viral titration.

When indicated, untreated or pre-treated cells with 1 µM or 5 µM MK-8722 (for Vero76 and Calu-3 cells, respectively) were inoculated at 4°C for 1 h to allow virus attachment to the cell membrane. The cells were washed three times in ice-cold PBS, and fresh D2, with or without MK-8722, was added before raising the temperature to 37°C for 1 h or 24 h for Vero76 and for 32 h for Calu-3 cells, as indicated. Cells were then harvested and processed for *N* RNA quantification as described below. For post-infection treatments, MK-8722 was added at 8 h post-infection (hpi) or 1 day post-infection (dpi) to reach indicated concentrations, controls receiving the same volume of D2.

### Quantification of infection by flow cytometry

Single-cell staining for viral protein and RNA by FISH-Flow was performed as described (39).

Briefly, cells were detached with trypsin 0.05% (Gibco 25300–024) for 10 min at 37°C and placed in 96-well plates. After centrifugation, cells were stained for 5 min on ice for viability (Viobility 405/452, Miltenyi 130–130-420, diluted 100 times in cold PBS). Cells were washed in PBS and fixed with PBS-4% PFA. Cells were washed in Perm Buffer and labeled for 30 min with anti-spike-AF488 antibody (R&D FAB105403G) diluted 250-fold in Perm Buffer and washed in Perm Buffer.

To further label viral RNA, cells were washed twice in hybridization wash buffer (HWB; 2× SSC, 10% formamide [MP Biomedicals FORMD002], and 0.2 mg/mL bovine serum albumin [BSA] UltraPure [ThermoFisher Scientific AM2618] in ultrapure water [Invitrogen 10977–035]), resuspended in hybridization buffer (10% [wt/vol] dextran sulfate [Calbiochem 265152], 1 mg yeast tRNA [Invitrogen AM2616], 2× SSC, 10% formamide, and 0.2 mg/mL BSA RNase-free in ultrapure water) with 50 nM total SARS-CoV-2-quasar670-specific FISH probes (Stellaris) in equimolar proportion, targeting regions of S, ORF1, and N genes, resuspended in hybridization buffer, and incubated for 12–16 h at 37°C. After three washes in HWB, cells were resuspended in PBS and analyzed by flow cytometry. The proportion of infected cells was quantified by flow cytometry (Guava 12-HT flow cytometer, Millipore) and analyzed using the native GuavaSoft 3.4 software.

### Western blotting

Cells were washed three times in PBS and lyzed with 1% Triton X-100 in 50 mM Tris pH 7.4, 150 mM NaCl, 1 mM EDTA, 1 mM EGTA, 10% glycerol, 1 mM dithiothreitol (DTT), 1% protease inhibitor cocktail (Sigma P8340), and 1% phosphatase inhibitor cocktail II and III (Sigma P5725 and P0044, respectively). Cell lysates were clarified by centrifugation at 15,000 g for 10 min at 4°C, aliquoted, and stored at −80°C. Protein content was quantified by BCA assay after sample thawing (Thermo Fischer J61522.AP). Around 25 µg protein was separated by SDS-PAGE 12% at 100 mV and transferred on nitrocellulose membranes in wet condition at 80 mA at 4°C for 3 h. Membranes were then saturated with TBS-0.5% Tween-20 (Sigma P1379, TBS-T) and 5% BSA. After three washes in TBS-T, membranes were incubated with primary antibodies described in Table 1 for 16 h at 4°C. After three washes in TBS-T, relevant secondary antibody conjugated to HRP (see Table 1) was added for 1 h at room temperature followed by washing three times in TBS-T. Finally, membranes were incubated with the West PicoPlus ECL HRP substrate (Thermo Fischer 34580). The signal was imaged with Fusion FX (Villber Lourmat) and quantified using Fiji ImageJ software. When indicated, data were computed from the Log2 fold change of the condition of interest over non-infected non-treated cells and presented as a heat map using GraphPad8.

**TABLE 1:** Antibodies used for western blotting

Target	Host and antibody nature	Conjugation	Supplier and reference	Dilution
β-actin	Goat polyclonal	–^*a*^	Abcam ab8229	4,000
p-Thr172-AMPK	Rabbit polyclonal	–	CST 2531	5,000
Pan-AMPK	Rabbit polyclonal	–	CST 2532	5,000
p-Ser79-ACC	Rabbit polyclonal	–	CST 11818	5,000
Pan-ACC	Rabbit polyclonal	–	CST 3676S	5,000
p-Ser555-ULK1	Rabbit polyclonal	–	CST 5869	5,000
Pan-ULK1	Rabbit polyclonal	–	CST 4773	5,000
p-Ser792-Raptor	Rabbit polyclonal	–	CST 2083	5,000
Nucleocapsid	Mouse IgG2b	Biotin	BIOSS bsm-41411M-biotin	3,000 (300 ng/mL)
Pan-Raptor	Rabbit polyclonal	–	CST 2280	5,000
SQSTM1/p62	Rabbit polyclonal	–	CST 5114	5,000
Goat	Monkey polyclonal	HRP	Promega V805A	5,000
Rabbit	Donkey polyclonal	HRP	Southern Biotech 6440–05	5,000
Streptavidin	–	HRP	Vector Laboratories, INC, Burlingame, CA, 94010	10,000

^*a*^ –, not conjugated.

### RNA extraction, N normalizer plasmid, and RT-qPCR

For RNA extraction, cells were washed three times in PBS and lysed in RA1 buffer. RNA was then extracted using the Nucleospin RNA kit (Macherey-Nagel 740955) following manufacturer instructions and stored at −80°C before RT-qPCR experiments. Primers and probes, designed according to original CDC recommendations (41), are described in Table 2 (Eurofins Genomics). The normalizer plasmid was obtained after T-A cloning of the corresponding cDNA using the High-Capacity RNA-to-cDNA kit (Thermo Fischer 387406) amplified using regular N-directed PCR with Platinum Taq (Thermo Fischer 10966034) following the TOPO cloning kit instruction (Thermo Fischer 10966034) and transformation of homemade thermocompetent TOP10 bacteria. Clones were selected using white-blue screening on Luria broth (LB) agar plates with 100 µg/mL ampicillin and 20 µg/mL X-Gal (Thermo Fisher R0941). Plasmids were extracted using the NucleoSpin Mini kit (Macherey Nagel 740588) and controlled by Sanger sequencing (Eurofins Genomics). For nucleocapsid RNA relative or absolute quantification, RT-qPCR was performed using 4 µL of cellular RNA and Taqman RNA-to-Ct 1-step kit (Thermo Fischer 4392938) following manufacturer recommendations using a LightCycler 480 (Roche). For IFN-I and ISG expression analysis, reverse transcription was first carried out using High-Capacity RNA-to-cDNA and further quantified using Sybr Master mix I (Roche #04707516001). Relative gene expression over *β-actin* in cell lysates was calculated by the 2^−ΔΔCt^ method, while absolute *N* abundance in supernatants was quantified using the N normalizer plasmid.

**TABLE 2:** Primer characteristics

Target	Name	Sequence	Tm (°C)
*Nucleocapsid*	N2-Fow	TTACAAACATTGGCCGCAAA	53.2
	N2-Rev	GCGCGACATTCCGAAGAA	56
	N2-Probe	ACAATTTGCCCCCAGCGCTTCAG	64.2
*β-Actin*	βAct-Fow	TGTTTGAGACCTTCAACACC	55.3
	βAct-Rev	ATGTCACGCACGATTTCC	53.7
	βAct-Probe	CCTCACCGAGCGCGGCTACAGCTTCA	72.9
*IFN-α*	IFN-α Fow	ACAACCTCCCAGGCACAAGGGCTGTATTT	72.5
	IFN-α Rev	TGATGGCAACCAGTTCCAGAAGGCTCAAG	71.2
*IFN-β*	IFN-β Fow	GTTCCTTAGGATTTCCACTCTGACTATGGTCC	68.6
	IFN-β Rev	GAACTTTGACATCCCTGAGGAGATTAAGCAGC	68.6
*Mx1*	Mx1 Fow	CTGGTGCTGAAACTGAAGAAAC	60.8
	Mx1 Rev	TACCTCTGAAGCATCCGAAATC	60.9
*OAS1*	OAS1 Fow	TCCACCTGCTTCACAGAACTACA	64.5
	OAS1 Rev	GGCGGATGAGGCTCTTGAG	63

### Immunofluorescence

Calu-3 cells were plated on Poly-L-Lysine (Sigma P8920; 0.1% in H_2_0) coated Ibidi slides (Ibidi 80841) at 1.2 × 10^5^/well for 5 days and infected with SARS-CoV-2 as described above. At 3 dpi, cells were washed, stained with 10 µg/mL Nile Red (Sigma 72485) in PBS at 37°C for 20 min, and washed prior to fixation when indicated. Cells were fixed in PBS-4% PFA for 1 h, washed, and permeabilized using 0.1% TritonX-100 in PBS for 10 min. Non-specific binding was blocked with 5% FCS in PBS for 30 min. Cells were further labeled in Perm Buffer with biotinylated-anti-N antibody at 300 ng/mL (BIOSS bsm-41411M-biotin) and/or anti-LAMP1 at 625 ng/mL (BD 555798), or anti-LC3b antibody at 1 µg/mL (Novus Biological, #NB600-1384SS) and anti-LAMP1 antibody at 625 ng/mL, overnight at 4°C and the relevant secondary for 1 h at room temperature (Streptavidin-AF488 Invitrogen S11223 at 2 µg/mL final, anti-mouse IgG1-Cy5 Abcam ab136127 at 1.5 µg/mL final or anti-Rabbit-Cy3 IgG at 1 µg/mL, Jackson 711–166-152, and anti-mouse IgG1-Cy5 at 1.5 µg/mL). After nuclear staining with 4′,6-diamidino-2-phenylindole (DAPI), cells were washed, and slides were mounted with Ibidi mounting medium (Ibidi 50001). Staining was observed by confocal microscopy (Xplore confocal microscope). At least five randomly chosen fields per condition were imaged and further analyzed using ImageJ software and the particle analysis function. LDs were identified as Nile Red-positive vesicular objects with a diameter >500 nm. Lysosomes were identified as LAMP1-positive vesicular objects with a diameter >700 nm. Analyses of lysosomes and LD was performed using *Z*-projections made from six consecutive slices 300 nm apart. The number of particles per infected cells, their mean area, and their intensity were then calculated. Colocalization on *z*-stack images was analyzed with the JACoP plugin using object-based colocalization and setting the threshold at 137, 134, and 120 for lysosomes, LD, and nucleocapsid objects, respectively. Manders’ overlap coefficients (MOCs) corresponding, respectively, to green objects colocalizing in red objects and inversely red objects colocalizing in green objects were computed.

### Lysosomal function analysis

Confluent Calu-3 cells in a 96-well plate were inoculated with either Alpha or Omicron variant at 0.05 MOI for 2 h, and cells were further cultured with fresh D2 up to 24 hpi. MK-8722 at the indicated concentration or D2 only was added for another 24 h. Lysosomes were then stained with 5 nM Lysotracker RED DND-99 (ThermoFisher L7528) in DMEM for 30 min following the manufacturer’s instructions. Cells were then detached with trypsin 0.05% for 10 min, transferred into a round bottom 96-well plate, stained for viability, and fixed with PBS-4% PFA. After 20 min, cells were washed, and the mean intensity of lysotracker staining was quantified by flow cytometry as described above.

### Evaluation of MK-8722 treatment on T cell activation

CD14-depleted PBMCs were thawed and cultured overnight at 37°C in R10 supplemented with 10 UI/mL IL-2 and 1 µg/mL DNAse (StemCell, 07900). Cells were then washed, resuspended cells in R10, and distributed at 10^5^ cells/well in a round bottom 96-well plate. Transact stimulation, which is a combination of activating anti-CD3 and anti-CD28 antibodies (Miltenyi, 130–111-160), consisted of 100-fold final dilution of Transact in R10. Alternatively, Spike or Nucleocapsid Peptivator T cell epitope peptide pools from either Spike or Nucleocapsid of SARS-CoV-2, respectively (Miltenyi 130–126-700 and 130–126-698, respectively), were used as stimulation sources at a final concentration of 500 ng/mL in R10. After stimulation for 30 min, Brefeldin A (5 µg/mL final) containing anti-CD107a-PE-Cy5 antibody (BD 555802) following the manufacturer’s instruction was added, and cells were incubated for 6 h. Cells were then washed and stained with anti-CD3-APC-H7 (BD 560176) and anti-CD4-APC (BD 551980) following manufacturer instructions for 10 min on ice, followed by viability staining as described above and fixation in PBS-4% PFA. Cells were further stained overnight with anti-IFNγ-PE (Miltenyi, 130–113-498) following the manufacturer’s instructions in Perm Buffer. Cells were washed in Perm Buffer and then in PBS. Frequencies of CD4^+^ and CD8^+^ T cells expressing CD107a^+^ or IFNγ^+^ were quantified by flow cytometry as described above. For each donor, we determined the net activation (NA) level for each marker after stimulation with Peptivator (NA [Peptivator]) by normalizing CD107a and IFNγ frequencies measured after stimulation with Peptivator over those measured after stimulation with Transact. This activation index reflects the capacity of the donor cells to respond to peptide-specific stimulation relative to its intrinsic stimulatory capacities.

### Evaluation of combined antiviral effects of MK-8722 and CD8^+^ T cells from vaccinated individuals on epithelial cell infection

CD8^+^ T cell activation is restricted to MHC-I; antigenic presentation occurs only between an antigen-presenting cell (i.e., infected cell) and the T cells with similar HLA. We first showed that Caco2 cells, unlike Calu-3 cells, express the MHC-I molecule HLA-A2 and were thus capable of presenting antigens to HLA-A2-restricted CD8^+^ T cells from vaccinated individuals. Therefore, confluent Caco2 cells were inoculated for 1 h with SARS-CoV-2 and further incubated for 8 h in the absence of virus. Previously amplified CD8^+^ T cells were thawed in R10 complemented with 5 IU/mL IL-2 to promote T cell survival, washed, and added to Caco2 cells at 8 hpi with or without 2.5 or 5 µM MK-8722. The co-cultures were further incubated at 37°C for 36 h. T cells were then collected and stained with anti-CD3-APC-H7 (BD 560176) and anti-CD8-APC (BD 345775) following the manufacturer’s instructions and for viability before fixation as described above. Cells were then intracellularly stained with anti-spike-AF488 (R&D FAB105403G) for 1 h at room temperature. Infection was monitored in CD3^−^CD8^−^ cells by flow cytometry as described above.

### Graphical representation and statistics

Data and graphs were computed using GraphPad Prism (San Diego, V8). If variables followed a normal distribution after Shapiro-Wilk, *t*-test or ANOVA was calculated; otherwise, statistical analyses were performed using the Mann-Whitney or Kruskal-Wallis test. *P*-values < 0.05 were considered significant. Tests were paired when experience design permitted.

## RESULTS

### MK-8722 inhibits SARS-CoV-2 replication in epithelial cells

**Fig 1:**
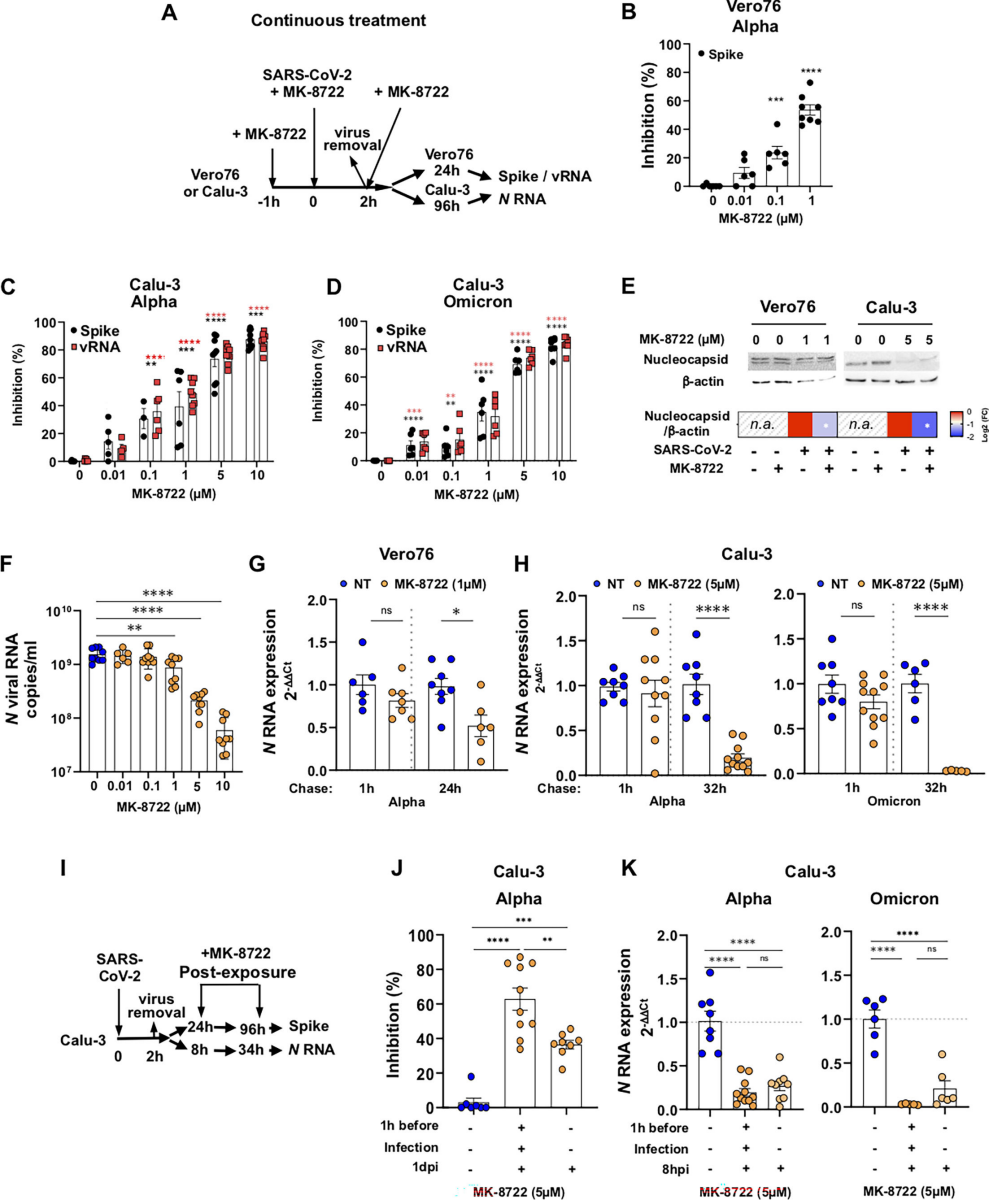
MK-8722 inhibits SARS-CoV-2 replication *in vitro. (*A) Schematic outline of the continuous treatment protocol with MK-8722. (B–D) Dose-dependent inhibition of Vero76 and Calu-3 cell infection with SARS-CoV-2 by continuous treatment with MK-8722. Inhibition of infection with the indicated SARS-CoV-2 variant in Vero76 was measured by flow cytometry (**B**) and that of Calu-3 by FISH-flow (**C and D**). *n* ≥ 5 independent experiments. Labeling for SARS-CoV-2 Spike (Black) and for viral RNA (Red). (E) Western blot of nucleocapsid and β-actin in infected cells after continuous treatment. Membranes of one representative experiment are shown. Heatmap represents the Log2 transform of the ratio over infected non-treated cells, quantified with ImageJ (*n* = 3 independent experiments). n.a.: non-applicable (as non-infected cells do not express N, see Fig. S2G). Student’s *t*-test: *P < 0.05 and **P < 0.01. (F*)* Dose-dependent inhibition of viral genome release from Calu-3 cells infected with the Alpha variant after continuous treatment with MK-8722 measured by quantification of *N* RNA in the supernatant by RT-qPCR. *n* ≥ 5 independent experiments. (G and H) Inhibition of viral replication after MK-8722 treatment*.* Cells were incubated with the indicated SARS-CoV-2 variant in the absence or the presence of the drug (1 µM for Vero76 [**F**] and 5 µM for Calu-3 cells [**G**]) for 1 h at 4°C to synchronize infection and chased for 1 h or for an additional 24 h in the case of Vero76 cells and for 32 h in the case of Calu-3 cells at 37°C. *N* RNA was quantified in cellular extracts by RT-qPCR. *n* ≥ 5 independent experiments. (I) Schematic representation of the post-exposure treatment protocol with MK-8722*.* (J and K) Post-exposure treatment with MK-8722 is sufficient to block infection by SARS-CoV-2*.* Cells were left untreated or treated with MK-8722 (5 µM) either continuously with Alpha variant or treated post-infection as in G. Infection was evaluated by quantification of Spike expression by flow cytometry (**I**) or by RT-qPCR using the 2^−ΔΔCt^ method (**J**). Values represent infection inhibition. *n* ≥ 6 independent experiments. Shown are mean ± SEM. Unless specified: ANOVA: * *P* < 0.05, **P < 0.01, ***P < 0.001, and ****P < 0.0001.

We first evaluated the activity of the pan-AMPK allosteric activator MK-8722 in Vero76 cells. As early as 1 h after treatment, MK-7288 activated AMPK and triggered downstream phosphorylation of ACC in a concentration-dependent manner from 0.01 to 1 µM (Fig. S1). The antiviral activity of MK-8722 was then monitored in Vero76 cells infected by SARS-CoV-2 as schematized (Fig. 1A). The drug was added at 0.01–1 µM to the cells 1 h before infection, during the inoculation with SARS-CoV-2 Alpha for 2 h and during the subsequent 24 h chase. At 1 dpi, Spike expression was quantified by flow cytometry (see gating strategy Fig. S2A). Spike^+^ cell frequency decreased in the presence of MK-8722, from 11.8% ± 0.7% in untreated to 5% ± 0.4% in 1 µM-treated conditions (ANOVA: *P* < 0.001, Fig. S2B), corresponding to an inhibition of infection of 53% ± 3% at 1 µM (ANOVA: *P* < 0.0001, Fig. 1B). MK-8722 antiviral activity was further analyzed in bronchial Calu-3 cells (see gating strategy Fig. S2C), which are more relevant to SARS-CoV-2 infection since they express TMPRSS2 and are competent for IFN-I signaling similar to bronchial cells *in vivo*, unlike Vero76 cells. Infection of Calu-3 cells with the Alpha variant in the presence of MK-8722 significantly decreased the frequency of viral Spike^+^ and viral RNA^+^ cells in a dose-dependent manner compared to untreated cells (Fig. S2D). At 10 µM MK-8722, the frequency of Spike^+^ and viral RNA^+^ cells decreased compared to untreated cells, from 30.8% ± 2.5% to 3.6% ± 0.2% and from 15.1% ± 1% to 1.5% ± 0.2%, respectively (ANOVA: *P* < 0.0001, Fig. S2D). MK-8722 similarly inhibited Omicron infection, the frequency of Spike^+^ and viral RNA^+^ cells decreasing compared to untreated cells (compare 27.5% ± 3% vs 4.5% ± 0.6% Spike+ cell frequency and 31% ± 3% vs 4.5% ± 0.4% RNA+ cell frequency at 10 µM and without MK-8722, respectively; Fig. S2E). These reductions were comparable to those induced by treating Calu-3 cells with the RNA-dependent RNA polymerase inhibitor Remdesivir at 1 µM (Fig. S2F). The IC50 of MK-8722 in Calu-3 cells against the Alpha variant was reached at 0.7 µM and an IC90 at 9 µM (*t*-test: *P* < 0.05; Fig. 1C). For the Omicron variant, MK-8827 showed an IC50 at 1.6 µM and an IC90 slightly above 10 µM (ANOVA: *P* < 0.05; Fig. 1D). Both RNA^+^ and Spike^+^ frequencies decreased after MK-8722 treatment. As MK-8722 antiviral activity against Alpha and Omicron variants is similar, the drug likely targets the same pathways in the two cases. Western blots were performed on Vero76 and Calu-3 lysates after infection and treatment. Their analyses are presented as a heatmap in which values are expressed in log2 fold changes (Log2[FC]), as described in the method section. As expected, the drug blocked infection, as shown by a significant decrease in N protein levels in both Vero76 and Calu-3 cells (−0.88 ± 0.2 and −1.7 ± 0.4 Log2[FC], respectively, *t*-test: *P* < 0.05 both, Fig. 1E; Fig. S2G). Furthermore, MK-8722 blocked viral production by Calu-3 cells in a concentration-dependent manner, as quantified by the decrease in *N* RNA by RT-qPCR from 1.5 ± 0.14 × 10^9^ to 6 ± 1.4 × 10^7^ viral copies/mL at 10 µM MK-8722 (ANOVA: *P* < 0.0001; Fig. 1F). MK-8722 did not alter the expression of ACE2 in Vero76 and Calu-3 cells, as measured by flow cytometry (Fig. S2H), nor impacted cell viability (Fig. S2I), indicative of a post-entry antiviral activity. Furthermore, we also investigated MK-8722 toxicity at 4 h, 24 h, and 96 h (Fig. S2J) and found that the drug was not toxic up to 10 µM. The 50% toxicity dose was calculated to be 57 µM at 96 h in Calu3 cells. This allows us to determine a therapeutic index of 76 against the SARS-CoV-2 Alpha variant and 36 against the Omicron variant, thus placing MK-8722 as an attractive antiviral candidate.

### MK-8722 exerts antiviral properties at the post-entry steps: post-infection treatment is sufficient to inhibit SARS-CoV-2 replication

We next investigated whether MK-8722 exerts its antiviral effect at the early infection steps. After MK-8722 pre-treatment of Vero76 and Calu-3 cells (at 1 µM and 5 µM, respectively), cells were inoculated with Alpha or Omicron variants for 1 h at 4°C to allow virus attachment. After virus removal, cells were incubated in the presence of the drug at 37°C to synchronize infection. Viral *N* RNA was quantified by RT-qPCR in the cells after a short (1 h) or long (24 h in Vero76 and 32 h in Calu-3 cells) chase times. As a control, cells were similarly infected but left untreated. Viral RNA accumulated after 1 h chase in the two cell types for the two variants similarly in treated and non-treated conditions, although the drug had a tendency to decrease relative *N* RNA levels in a non-significant statistical manner (1 ± 0.12 vs 0.82 ± 0.08 for Vero76, 1 ± 0.05 vs 0.91 ± 0.15 and 1 ± 0.1 vs 0.8 ± 0.08 for Alpha and Omicron variants in Calu-3 cells, respectively, *t*-test: ns in all conditions; Fig. 1G and H). In contrast and as expected, *N* RNA expression was significantly reduced after a longer chase (1 ± 0.09 vs 0.52 ± 0.12 for Vero76, 1 ± 0.11 vs 0.2 ± 0.04 and 1 ± 0.1 vs 0.03 ± 0.003 for Alpha and Omicron variants in Calu-3 cells, respectively, *t*-test *P* < 0.05, <0.0001, and <0.0001, respectively). These results suggest that MK-8722 has little antiviral effect during the early steps of infection, namely adsorption and fusion, but acts in a mechanism controlling post-entry replication steps.

We next evaluated if MK-8722 was still active once cells had been infected (Fig. 1I). Post-exposure treatment at 1 dpi and until harvest at 3 dpi efficiently blocked viral replication, quantified by Spike expression by 36% ± 7% (Fig. 1J, ANOVA: *P* < 0.0001), although still less than the continuous treatment, which reduced infection by 63% ± 6% (ANOVA: *P* < 0.0001). Continuous and post-exposure treatment with MK-8722 (5 µM) resulted in a similar inhibition of viral genome accumulation of both Omicron and Alpha variants in infected cells (Fig. 1K). Indeed, after continuous treatment with MK-8722 for 34 h, *N* RNA relative amount decreased significantly by 80% ± 0.04% and 97% ± 0.7% in Alpha and Omicron infected cells, respectively (*t*-test: *P* < 0.0001 for both variants vs untreated cells). Limiting the MK-8722 treatment to the post-exposure phase, namely from 8 hpi, decreased *N* RNA relative amount by 75% ± 0.05% and 80% ± 20% in Alpha and Omicron infected cells, respectively (ANOVA: *P* < 0.0001 for both Alpha and Omicron variants vs untreated cells). Altogether, these data suggest that post-exposure treatment by MK-8722 is sufficient to inhibit viral infection.

### MK-8722-induced AMPK activation inhibits lipid metabolism to control SARS-CoV-2 replication

**Fig 2:**
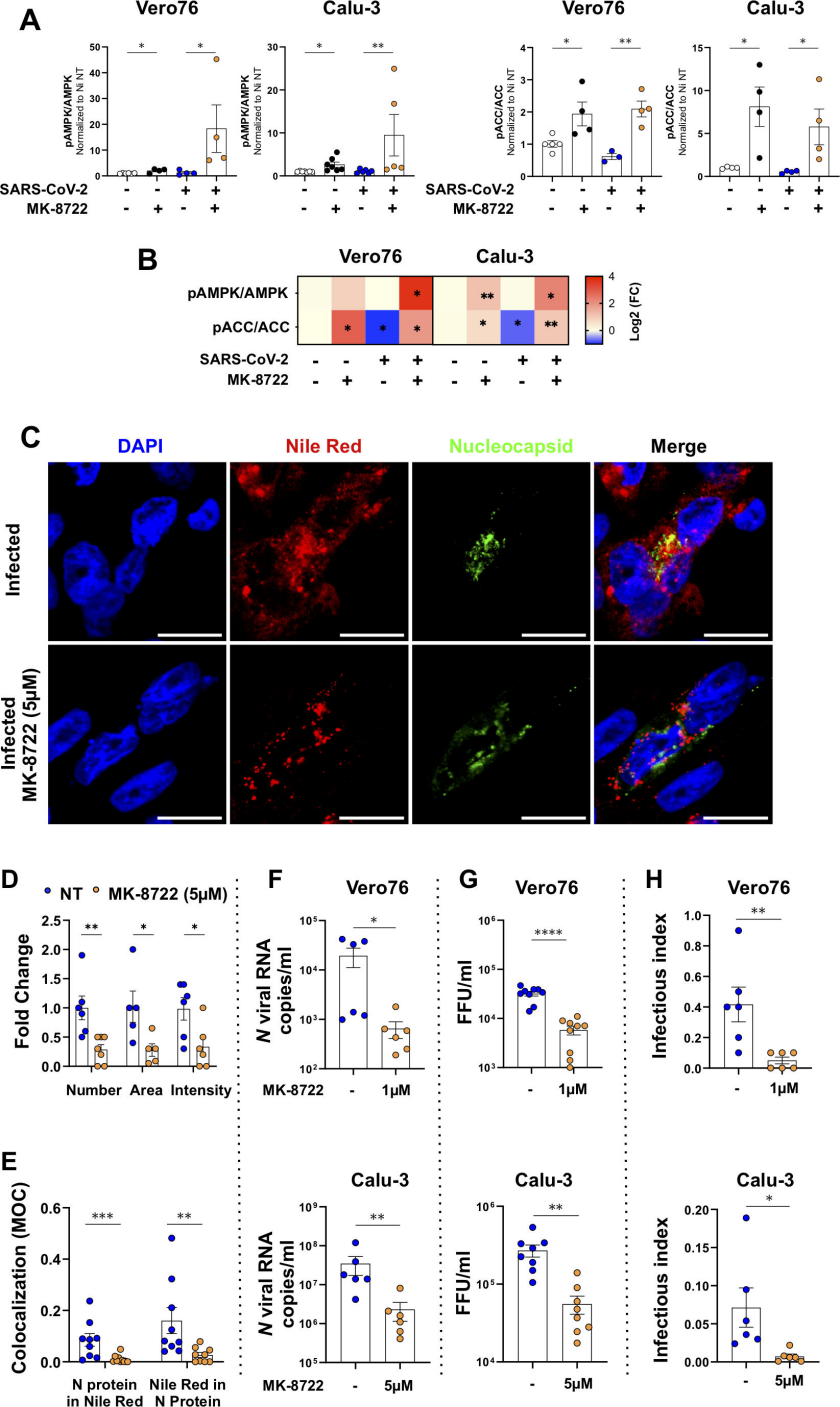
Inhibition of SARS-CoV-2 replication by MK-8722 is associated with a reduction of ipid droplets and release of defective viral particles. (A) The activation ratio of AMPK and ACC was analyzed by western blot in Vero76 and Calu-3 cells after continuous MK-8722 treatment as indicated in Fig. 1A western blots were quantified by ImageJ, and each marker value in the indicated condition was normalized over the corresponding marker expression value in the non-infected non-treated condition. Shown are mean ± SEM. *n* = 2 independent experiments. (B) Quantification of the western blot presented as heat maps*:* heat maps represent the transformation by Log2 of the expression/phosphorylation values quantified on each blot by ImageJ relative to corresponding non-infected non-treated conditions for Vero76 and Calu-3 cells. *n* ≥ 2 independent experiments. Student’s *t*–test: *P < 0.05 and **P < 0.01. (C) Morphological analyses*.* Calu-3 cells were infected with the SARS-CoV-2 Alpha variant in the presence of continuous treatment with MK-8722 (5 µM) or not. At 3 dpi, lipid droplets, defined as indicated in the method section, were stained with Nile Red lipophilic dye before cell fixation. Nucleoprotein protein (Green) expression detected by immunofluorescence, Nile Red (red) staining, and nuclei staining with DAPI (blue) was observed by confocal microscopy. One representative field out of five observed per condition is presented. Bar: 20 µm. Shown are images representative of *n* = 3 independent experiments. (D) Quantitative analysis of images shown in A using ImageJ of the number, area, and intensity of LDs, as defined in the method section within infected cells, characterized by the expression of nucleocapsid protein. Values were normalized to the corresponding infected non-treated conditions. *n* = 3 independent experiments. (E) LDs and N-protein colocalization. The fraction of LDs colocalizing in nucleocapsid staining and inversely was quantified using the JACoP plugin from ImageJ software, and corresponding MOCs are presented. (F) Viral genomes released. Cells were infected with the Alpha SARS-CoV-2 variant in the absence or in the presence of continuous treatment with MK-8722 at the indicated concentrations. Viral *N* RNA released by Vero76 cells at 1 dpi (upper panel) and Calu-3 cells at 4 dpi (lower panel) cells was quantified by RT-qPCR. *n* ≥ 3 independent experiments. (G) Viral titers released by Vero76 (upper panel) and Calu-3 (lower panel) cells treated as above in (**D**). *n* = 3 independent experiments. (H) The infectious index of released viruses was determined as described in the methods section using matched viral titers and viral copy number values Vero76 (upper panel) and Calu-3 (lower panel) cells obtained as in (**D**) and (**E**). *n* = 4 independent experiments. Student’s *t*-test and Mann-Whitney * *P* < 0.05, **P < 0.01, ***P < 0.001, and ****P < 0.0001.

We further examined the mechanism of SARS-CoV-2 inhibition by MK-8722. Quantification of the western blots relative to non-infected non-treated control condition is shown in Fig. 2A and summarized in a heat map in which values were expressed as Log2(FC) (Fig. 2B). As expected, MK-8722 treatment of both Vero76 and Calu-3 cells significantly activated AMPK that became phosphorylated at position Thr172 (0.85 ± 0.4 and 1.2 ± 0.3 log2[FC], *t*-test: *P* < 0.05 and *P* < 0.01, respectively; Fig. 2A and B; Fig. S3A and S3B). AMPK phosphorylation was comparable in infected and non-infected cells (0.0 ± 0.5 and −0.02 ± 0.3 log2[FC], respectively), while MK-8722 treatment stimulated AMPK phosphorylation even more in infected than in non-infected cells (3.1 ± 0.4 and 2.3 ± 0.9 log2[FC], respectively, *t*-test: *P* < 0.05 both), which confirms that MK-8722 inhibits SARS-CoV-2 infections through AMPK activation. As AMPK is a major regulator of lipid and protein metabolism, therefore, we investigated during SARS-CoV-2 infection of Vero76 and Calu-3 cells the phosphorylation level of ACC and Raptor, which are directly downstream of AMPK. Interestingly, the marker of protein synthesis inhibition S792 Raptor phosphorylation remained unchanged regardless of the conditions (Fig. S3A and S3B), indicating that the MK-8722 antiviral effect did not rely on Raptor-mediated translational shutdown. However, SARS-CoV-2 infection strongly decreased S79 ACC phosphorylation significantly (−1 ± 0.29 and −0.7 ± 0.2 Log2[FC], respectively and *t*-test: *P* < 0.05, Fig. 2A and B; Fig. S3A and B), while it was significantly increased by MK-8722 treatment during infection (1.9 ± 0.5 and 1 ± 0.2 Log2[FC], *t*-test: *P* < 0.05 and *P* < 0.01, respectively; Fig. 2A and B). These data indicate that MK-8722-induced AMPK activation disrupts lipid metabolism through the inhibition of ACC, which mitigates the establishment of a virus-favorable lipid environment.

We next explored the impact of MK-8722 on the distribution of lipids associated with viral factories. Therefore, lipid reorganization in infected Calu-3 cells was monitored after continuous treatment with MK-8722 by staining lipids with the lipophilic marker Nile Red analyzed by confocal microscopy. As expected (35), treatment with MK-8722 in uninfected cells blocked almost entirely the synthesis of lipids, as shown by the loss of Nile Red staining (Fig. S3C). Infection increased the overall Nile Red staining by 40% ± 10% compared to non-infected cells (Fig. S3D, ANOVA: *P* < 0.05) but also dramatically modified Nile Red cellular distribution into aggregates likely corresponding to lipid droplets (compare Fig. 2C; Fig. S3C). This overall infection-induced increase in Nile Red staining was abolished by MK-8722 treatment (Fig. S3D, 0.943-fold ± 0.14-fold change, infected non-treated vs treated cells, ANOVA: *P* < 0.05) and returned to untreated uninfected cell level. Furthermore, in infected cells, identified by viral N protein labeling, MK-8722 significantly reduced the number, area, and intensity of lipid droplets (as defined in the method section; Fig. 2C and D, 0.29 ± 0.09 and 0.28 ± 0.1 and 0.33 ± 0.15 fold-change over infected non-treated cells, *t*-test: *P* < 0.01, *P* < 0.05 and *P* < 0.05, respectively). We then evaluated if the N protein, a marker of virus replication in viral factories, was redistributed out of lipid droplets by the drug. In the absence of MK treatment, N protein and lipids colocalized as expected, although partially, with MOC being in the range of previous reports (13). This colocalization decreased significantly in cells infected in the presence of MK-8722, as indicated by lower MOCs for N protein in Nile Red (0.085 ± 0.024 vs 0.01 ± 0.005, Mann-Whitney *P* < 0.001) and for Nile Red in N protein (0.16 ± 0.05 vs 0.027 ± 0.01, Mann-Whitney *P* < 0.01) in MK-8722 treated vs untreated infected cells (Fig. 2C and E). Altogether, SARS-CoV-2 infection increases the accumulation of lipid droplets with which viral components colocalize. MK-8722 treatment reverses these effects, likely reflecting an alteration of cell lipid composition following increased ACC phosphorylation, thus limiting the production of viral factories.

As SARS-CoV-2 is an enveloped virus, modification in cellular lipid metabolism modification could alter the lipid composition of the viral membrane and, in turn, viral particle infectivity (6, 8–12). We thus measured viral production by infected Vero76 and Calu-3 cells by quantification of secreted viral nucleocapsid *N* RNA by RT-qPCR. In line with the reduction of secreted viral *N* RNA in the presence of the drug shown above (Fig. 1F), the level of viral *N* RNA secreted by both infected cell lines was reduced by 10-fold upon continuous MK-8722 treatment (Fig. 2F, 2 ± 0.8 × 10^4^ vs 6 ± 2 × 10^2^ and 3.4 ± 1.7 × 10^7^ vs 2.3 ± 1.2 × 10^6^ RNA copies/mL for Vero [upper] and Calu-3 cells [lower], respectively, *t*-test: *P* < 0.05 for Vero76 and *P* < 0.01 for Calu-3 cells). Accordingly, the amount of infectious viruses released by infected cells titrated in foci forming assay was statistically decreased by MK-8722 in Vero76 (from 3 ± 0.3 × 10^4^ to 5.8 ± 1 × 10^3^ FFU/mL) and Calu-3 cells (from 2.6 ± 0.5 × 10^5^ to 5.5 ± 1 × 10^4^ FFU/mL, *t*-test: *P* < 0.05 for both cell types; Fig. 2G)

We next calculated an infectious index of released particles (described in the method section), which inversely correlates with virus infectivity. The drug reduced the frequency of newly produced infectious viruses by both cell lines (infectious index: 0.41 ± 0.11 vs 0.05 ± 0.02 in Vero76 cells [Mann-Whitney *P* < 0.05], and 0.07 ± 0.03 vs 0.007 ± 0.003 in Calu-3 cells [Mann-Whitney *P* < 0.05] in the presence and absence of the drug, Fig. 2H). This inhibitory effect of MK-8722 on the production of infectious particles could not be explained by a direct inhibitory role of MK-8722 present in the culture media on foci formation. Indeed, tested cell culture media were highly diluted in the assay (at least 1,000 times) corresponding to an MK-8722 concentration <0.005 µM, which is far under the limit of the efficacy of MK-8722 in Vero76 cells (Fig. 1B; Fig. S2B). Furthermore, MK-8722 exposure was limited to the 2 h inoculation. Our data suggest that the lipid metabolism alteration induced by MK-8722 counteracts SARS-CoV-2 replication and affects the production and infectivity of released viral particles.

### Autophagy induction upon MK-8722 treatment promotes lysosomal degradation of viral components

**Fig 3:**
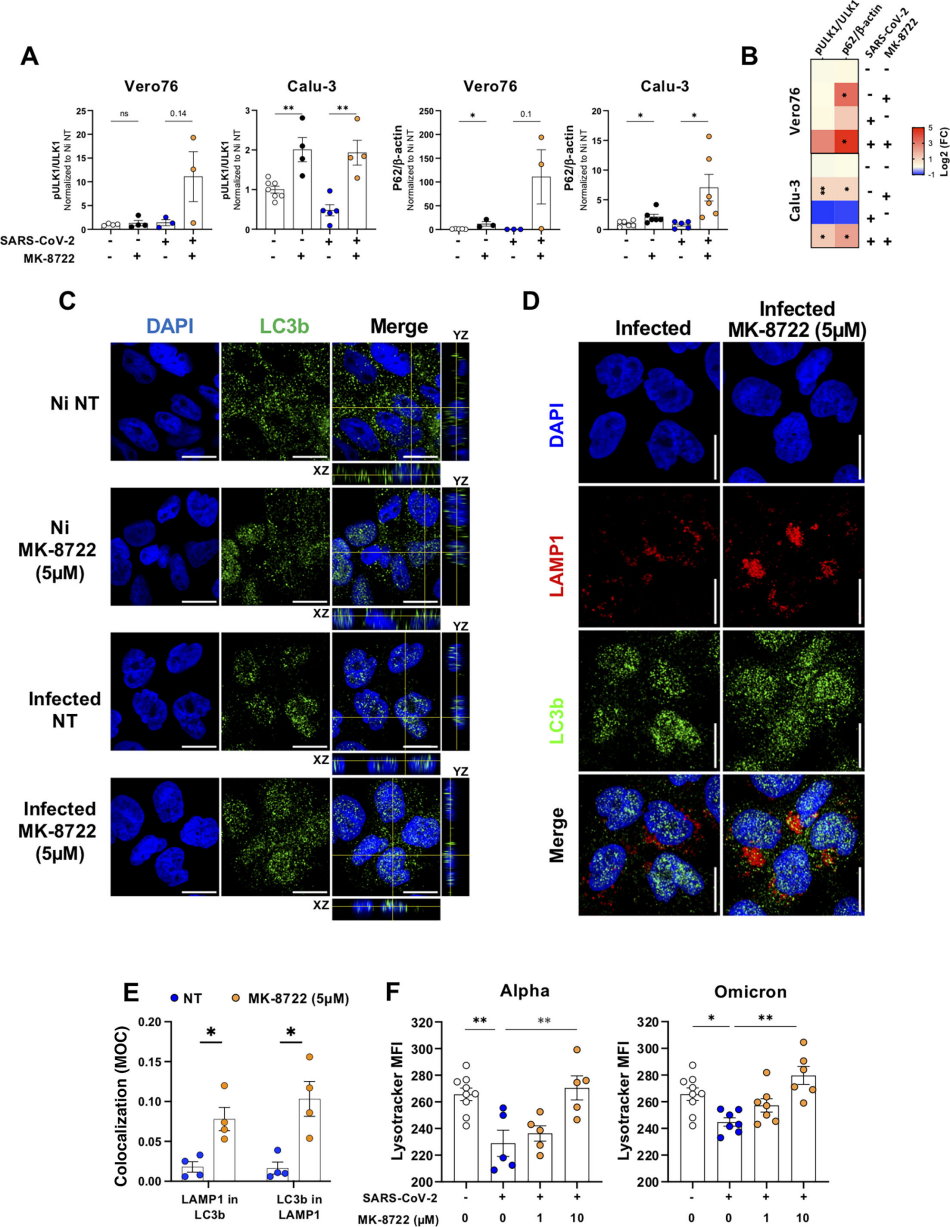
MK-8722 treatment increases the autophagic flux and lysosomal activity. (A) Activation ratio of ULK1 and expression of p62 analyzed by western blot: Vero76 and Calu-3 cells were analyzed by western blot after continuous MK-8722 treatment as indicated in Fig. 1A. Western blots were quantified by ImageJ, and each marker value in the indicated condition was normalized over the corresponding marker expression value in the non-infected non-treated condition. Shown are mean ± SEM. *n* = 2 independent experiments. (B) Quantification of the western blot presented as heat maps: heat maps represent the transformation by Log2 of the expression/phosphorylation values quantified on each blot by ImageJ relative to corresponding non-infected non-treated conditions for Vero76 and Calu-3 cells. *n* ≥ 2 independent experiments. Student’s *t*-test: *P < 0.05 and **P < 0.01. (C) Distribution of LC3b upon infection and MK-8722 treatment. Calu-3 cells were infected or not with the SARS-CoV-2 Alpha variant in the presence of continuous treatment with MK-8722 (5 µM) or not. At 3 dpi, cells were then fixed and LC3b (green) was labeled by immunofluorescence. Nuclei were stained with DAPI (blue). Cells were observed by confocal microscopy. One representative slice of each field out of four observed per condition is presented. Bar: 20 µm. Yellow lines represent the section cut for orthogonal view (XZ across and YZ down). Images representative of *n* = 3 independent experiments (D) Distribution of LC3b relative to LAMP1 upon infection and MK-8722 treatment: Calu-3 infected cells, in the presence of continuous treatment with MK-8722 (5 µM) or not (same field as shown in C), were additionally labeled for LAMP-1 (red) and observed by confocal microscopy. Nuclei were stained with DAPI (blue). Cells were observed by confocal microscopy. One representative field out of four observed per condition is presented. Bar: 20 µm. Images representative of *n* = 3 independent experiments. (E) LAMP1 and LC3b protein colocalization. The fraction of lysosomal objects colocalizing with LC3b labeling foci and inversely was quantified using the JACoP plugin from ImageJ software, and corresponding MOCs are presented. *n* = 3 independent experiments. (F) Lysosomal activity*.* Calu-3 cells were infected with the indicated SARS-CoV-2 variant in the presence of indicated MK-8722 concentration added at 24 hpi and cultured for another 24 h. Lysosomal activity was then analyzed using lysotracker DND99 dye and quantified by flow cytometry. Shown are lysotracker MFI collected from *n* ≥ 5,000 cells analyzed. *n* = 3 experiments. ANOVA and Mann-Whitney * *P* < 0.05, **P < 0.01, ***P < 0.001, and ****P < 0.0001.

Since autophagy is also downstream of AMPK activation, we monitored S555 ULK1 phosphorylation and p62 relative expression as markers of the initiation of autophagy by western blot. While neither MK-8722 nor SARS-CoV-2 infection alone affected ULK1 phosphorylation (with the exception of Calu-3 cells, non-infected but treated, where the increase reached 1 ± 0.2 Log2[FC], *P* < 0.01), MK-8722 treatment during infection was associated with an increase in ULK1 S555 phosphorylation in both cell types (from 0.05 ± 0.5 to 2.8 ± 1.2 Log2[FC] in Vero76 cells and from −0.9 ± 0.5 to 1.3 ± 0.6 Log2[FC] in Calu-3 cells, *t*-test: ns and *P* < 0.05, respectively; Fig. 3A and B; Fig. S3A). Concomitantly, p62 expression strongly and significantly increased in both cell types (3.33 ± 0.56 and 2 ± 0.6 Log2[FC] for Vero76 and Calu-3 cells, respectively, *t*-test *P* < 0.05; Fig. 3A and B). Increased p62 levels are most likely due to the transcriptional response to AMPK-dependent activation of Nrf2 upon MK-8722 treatment (42), which could be further increased by oxidative stress upon SARS-CoV-2 infection. In addition, to dissect whether p62 accumulation is associated with *de novo* induction of autophagy or rather a block of autophagic flux, we examined the impact of MK-8722 on lysosomal-autophagosomal fusion by colocalization of the lysosomal marker LAMP1 and the autophagosome marker LC3b. Calu-3 cells were infected by SARS-CoV-2 without and with MK-8722 (5 uM), double labeled with LC3b and LAMP1, and colocalization of the two markers quantified by confocal microscopy (Fig. 3C through E). We first analyzed the distribution of LC3b and observed that in uninfected control untreated cells, LC3b was only localized in the cytoplasm (Fig. 3C, top), whereas treatment with MK-8722 resulted in a partial redistribution of LC3b into the nucleus as clearly visualized by the overlap of nuclear and LC3b signals in XZ and YZ sections (Fig. 3C, second row). SARS-CoV-2 infection induced an almost complete nuclear translocation of LC3b (Fig. 3C, third row), whereas MK-8722 treatment upon infection restricted the nuclear distribution of LC3b to the levels of uninfected MK-8722-treated cells (Fig. 3C, bottom). Nuclear localization of LC3b has previously been described following starvation or metabolic stress. Accordingly, our results confirm that SARS-CoV-2 infection induces metabolic stress in the cells (6, 13, 15, 19) that is limited by MK-8722 treatment. We then compared the distribution of LC3b and LAMP. MK-8722 treatment, compared with no treatment, increased the accumulation of LC3b-positive structure in the LAMP1 compartment as shown by the increased MOCs in treated vs non-treated-infected cells (LAMP1 signal in LC3b signal 0.078 ± 0.014 vs 0.01 ± 0.006, Mann-Whitney *P* < 0.05, and LC3b in LAMP1 signal 0.04 ± 0.02 vs 0.015 ± 0.007 Mann-Whitney *P* < 0.05), showing that autophagic flux is amplified (Fig. 3D and E). These results indicate that MK-8722 restored the autophagic flux that had been interrupted by SARS-CoV-2 infection in Calu3 cells. We then investigated whether increased autophagic flux resulted in increased lysosomal activity using lysotracker staining. After infection for 24 h, cells were treated with MK-8722 for a further 24 h, and lysotracker staining in Calu-3 was analyzed by flow cytometry. MK-8722 increased lysosomal activity similarly after infection by Alpha and Omicron variants (lysotracker mean fluorescence intensity [MFI]: 286 ± 12 and 289 ± 9.6 in MK-8722 treated vs 253 ± 34 and 251 ± 6 in non-treated cells, respectively, for each variant, ANOVA: *P* < 0.05 at least; Fig. 3F).

**Fig 4:**
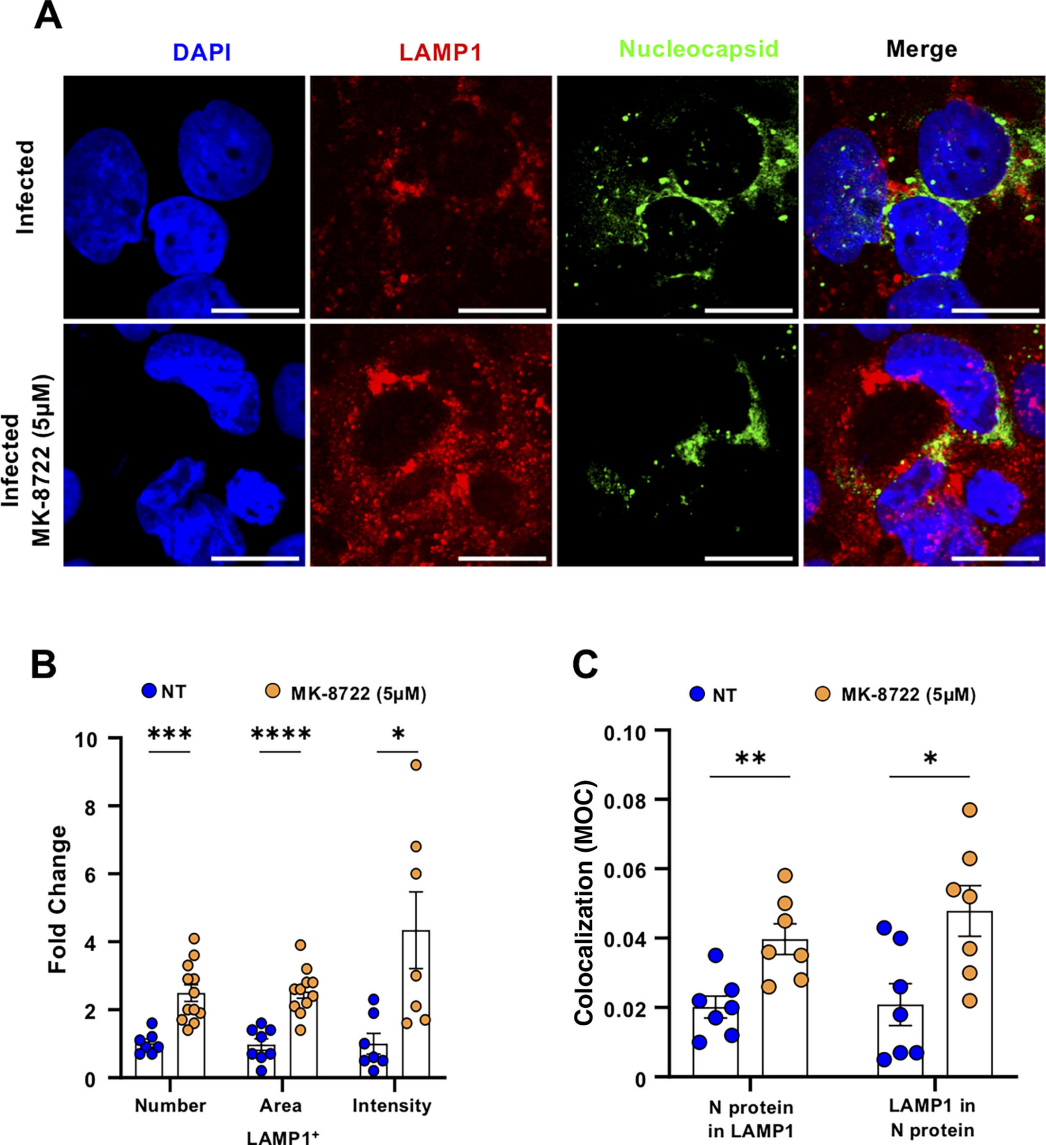
MK-8722 targets viral components to lysosomes. (A) Morphological analysis: Calu-3 cells were infected with the SARS-CoV-2 Alpha variant in the presence of continuous treatment with MK-8722 (5 µM) or not. At 3 dpi, cells were then fixed, and LAMP1 (red) and Nucleocapsid N (green) protein expression were detected by immunofluorescence. Nuclei were stained with DAPI (blue). Cells were observed by confocal microscopy. One representative field out of five observed per condition is presented. Bar: 20 µm. Images representative of *n* = 3 independent experiments. (B) Quantitative analysis of images shown in C using ImageJ of number, area, and intensity of lysosomes, identified as defined in the methods section, within infected cells, characterized by the expression of nucleocapsid protein. Values were normalized to the infected non-treated conditions. *n* = 3 independent experiments. (C) LAMP1 and N protein colocalization. The fraction of lysosomal objects colocalizing with nucleocapsid staining and inversely were quantified using the JACoP plugin from ImageJ software, and corresponding MOCs are presented. *n* = 3 independent experiments. ANOVA and Mann-Whitney * *P* < 0.05, **P < 0.01, ***P < 0.001, and ****P < 0.0001.

We then asked whether restoration of autophagic flux and increased lysosomal activity by MK-8722 could result in targeting viral proteins to lysosomes. We first evaluated the lysosome distribution after MK-8722 treatment in infected cells studying LAMP1 distribution in Calu-3 cell by immunolabeling and confocal microscopy (Fig. 4A; Fig. S4B). SARS-CoV-2 infection significantly decreased LAMP1 relative expression in Calu-3 cells as expected (Fig. 4A; Fig. S4B**,** quantified in Fig. S4C as 0.47 ± 0.06 fold change, ANOVA: *P* < 0.05). This decrease likely reduced LAMP1^+^ lysosome size as infection inhibits autophagosome-lysosome fusion (23, 25, 26). MK-8722 treatment during infection significantly restored LAMP1 relative expression to its level in non-infected non-treated condition (1.15 ± 0.11 fold change, ANOVA: *P* < 0.01 compared to infected non-treated condition; Fig. S4C). Further evaluation of LAMP1^+^ lysosome parameters in infected cells (identified by the expression of viral protein N), as defined in the method section, showed that MK-8722 significantly increased LAMP1^+^ lysosome number, area, and specific intensity, indicative of their enhanced activity (Fig. 4B; 2.5 ± 0.25 and 2.53 ± 0.2 and 4.3 ± 1.1 fold change over infected non-treated cells, *t*-test: *P* < 0.001, *P* < 0.05, and *P* < 0.0001, respectively). When colocalization of viral N protein and LAMP1 was quantified in infected cells, MK-8722 treatment resulted in higher MOC as compared to infected non-treated cells (N protein in LAMP1 signal: 0.02 ± 0.003 vs 0.05 ± 0.004, Mann-Whitney *P* < 0.01; and LAMP1 in N protein signal: 0.021 ± 0.006 vs 0.048 ± 0.007, Mann-Whitney *P* < 0.05; Fig. 4C). As the N protein is known to be associated with replication complexes, our results indicate (43) that MK-8722 restores the autophagic flux to address viral replication complexes and/or fully formed viral particles to the lysosome, where they are degraded.

Altogether, these results suggest that the antiviral mechanism of MK-8722 relies for one part on the increased ACC phosphorylation. This decreases lipid synthesis, which may reduce lipid droplet content and thus affect the establishment of viral factories. A switch in lipid metabolism also translates into the generation of more defective viral particles, reducing infection spreading. As a complementary anti-viral mechanism, the AMPK activation-dependent restoration of the autophagic flux, which is otherwise blocked by SARS-CoV-2 proteins due to ULK1, plays a role. Phosphorylation of ULK1 and increased p62 expression likely result in increased access of viral components to phagosomes and further phagosome fusion with lysosomes, resulting in subsequent degradation of viral proteins in phagolysosomes, preventing viral dissemination.

### MK-8722 treatment promotes innate and cellular antiviral immunity

**Fig 5:**
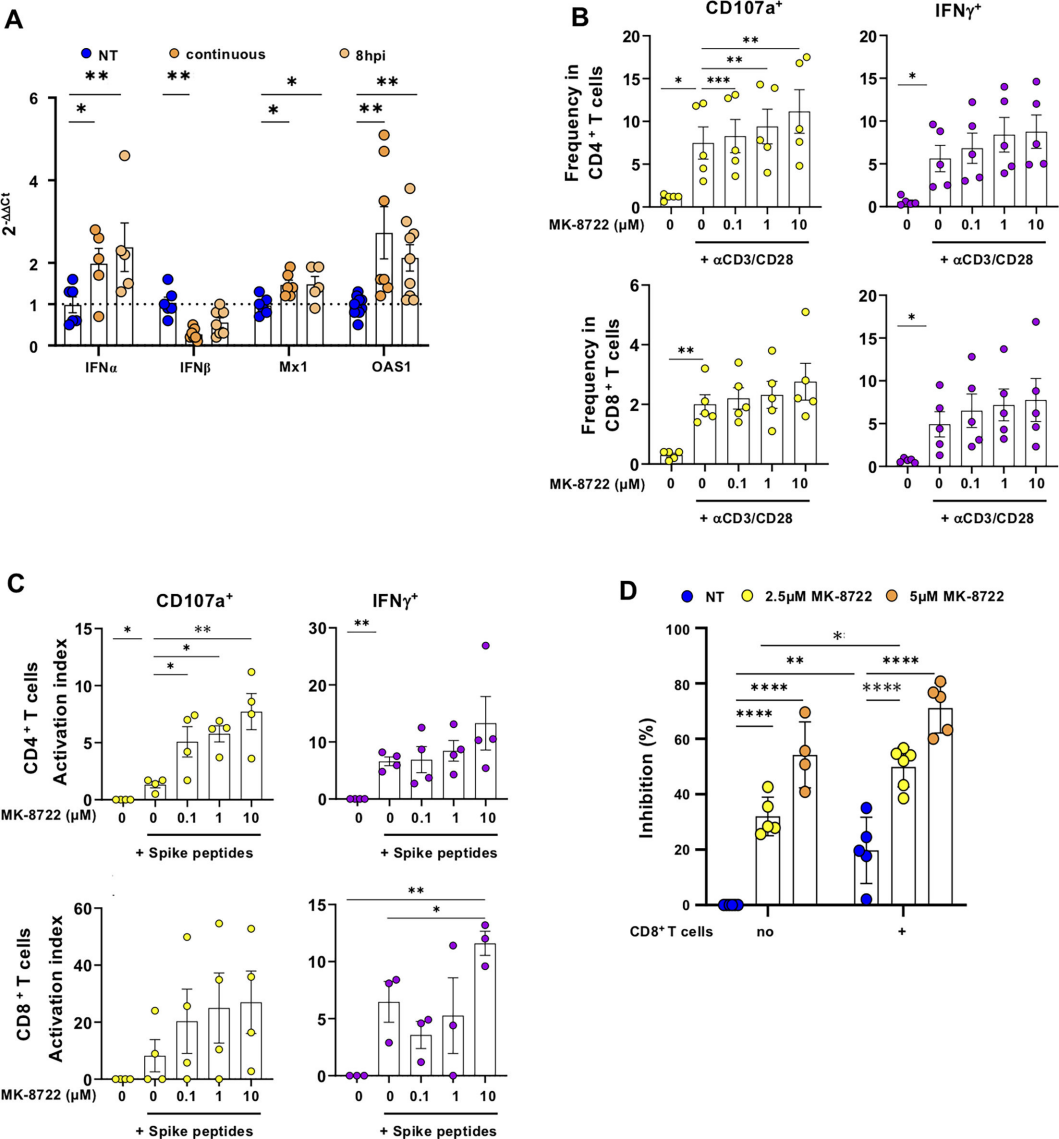
Restoration of intrinsic and cellular immunity against SARS-CoV-2 after MK-8722 treatment. (A) IFN-I response. Calu-3 cells were infected with SARS-CoV-2 Alpha variant in the presence of MK-8722 (5 µM) continuous treatment (dark orange) or initiated at 8 hpi (light orange) or without treatment (blue). IFN-I response was evaluated by quantifying *IFNα*, *INFβ*, *Mx1*, and *OAS1* mRNA by RT-qPCR of indicated genes at 1 dpi. Data represent the expression of each mRNA relative to *β-actin* mRNA levels and expressed following the 2^−ΔΔCt^ method. *n* = 3 independent experiments. (B) PBMCs from healthy SARS-CoV-2 vaccinated donors depleted in CD14 cells were stimulated for 6 h with anti-CD3 and anti-CD28 antibodies in the absence or in the presence of MK-8722 (5 µM). IFNγ^+^ and CD107a^+^ cell frequencies within CD4^+^ and CD8^+^ T cell populations were evaluated by flow cytometry. *n* = 5 independent donors analyzed in independent experiments. (C) CD14-depleted PBMCs from corresponding donors in (**A**) were stimulated for 6 h with a T cell SARS-CoV-2 spike peptide pool (Peptivator, Miltenyi), and IFNγ^+^ and CD107a^+^ cell frequencies were quantified as in (**A**). Activation levels are presented as activation index calculated as described in the methods section. n ≥ 3 independent donors analyzed in independent experiments. (D) Caco2 cells were infected 2 h with the Alpha variant of SARS-CoV-2 and treated post-exposure (8 hpi) with MK-8722 (5 µM) alone or together with CD8^+^ T cells from SARS-CoV-2-vaccinated individuals. Infection was evaluated by quantification of Spike expression in cells at 2 dpi by flow cytometry and presented as inhibition of Spike expression frequency. *n*=5 independent donors performed in independent experiments. Data presented are mean ± SEM. ANOVA and *t*-test: *P < 0.05, **P < 0.01, ***P < 0.001, and ****P < 0.0001.

The lack of type-I IFN response is a major immune parameter contributing to COVID-19 disease (30, 44, 45). Limiting viral factories and completion of autophagy will most likely result in the degradation of viral proteins, which escape RIG-I sensing, thereby restoring IFNα and IFNβ production and associated downstream ISG such as OAS1 and Mx1. Thus, we investigated the IFN-I response in infected Calu-3 cells after continuous or post-infection treatment with MK-8722 (Fig. 5A). Compared to the untreated condition, continuous and post-infection treatment with MK-8722 increased *IFNα* mRNA expression by 100% ± 38% and 140% ± 58% (Kruskal-Wallis: *P* < 0.05 for both treatments) while that of *IFNβ* decreased by 73% ± 5% and 45% ± 11% for continuous and post-infection treatment, respectively (Kruskal-Wallis: *P* < 0.01 and ns, respectively). Regarding ISG mRNA expression, *Mx1* level increased by 1.46-fold ± 0.12-fold and 1.49-fold ± 0.19-fold for continuous and post-infection treatment, respectively (Kruskal-Wallis: <0.05 both, respectively), while *OAS1* mRNA expression increased by more than twofold in all treated conditions (2.8-fold ± 0.6-fold and 2.1-fold ± 0.3-fold, respectively, Kruskal-Wallis: *P* < 0.01). Altogether, our results suggest that even post-exposure MK-8722 treatment can drive early IFN-I expression and downstream ISG, which, by controlling infection, could limit at least partially severe outcomes in patients (27, 28, 30, 44, 46).

The T cell response is crucial to control SARS-CoV-2 infection (47–49) and relies on the metabolism reprogramming of the effector cell. Therefore, we investigated the impact of MK-8722-mediated AMPK activation on the T cell response using primary cells obtained from the peripheral blood of healthy individuals. We found that MK-8722 alone compared to carrier DMSO at identical concentrations did not activate T cells from healthy donors (Fig. S6A). We then investigated if MK-8722 exposure affected T cell responses upon T cell receptor (TCR) engagement. Healthy donor PBMCs were stimulated with anti-CD3 and anti-CD28 (CD3/CD28, TransAct, Fig. S6B) to mimic TCR engagement and treated with MK-8722 for 6 h. Intracellular IFNγ^+^ and CD107a^+^ frequencies were then analyzed in CD4^+^ and CD8^+^ T cells. As expected, CD3/CD28 stimulation increased both IFNγ^+^ and CD107a^+^ frequencies in both CD4^+^ and CD8^+^ T cells (Fig. 5B; 5.6% ± 1.5% and 7.5% ± 1.9% vs 0.6% ± 0.2% and 1.1% ± 0.2% for CD4^+^ T cells, and 4.9% ± 1.5% and 2% ± 0.3% vs 0.68% ± 0.1% and 0.3% ± 0.06% for CD8^+^ T cells, for IFNγ^+^ and CD107a^+^ frequencies, respectively, *t*-test: *P* < 0.05 at least). The concentrations of MK-8722 tested had no negative impact on the expression of activation markers and even showed a trend toward increased activation with MK-8722 treatment. These results indicate that MK-8722 does not affect degranulation and IFNγ responses and is thus compatible with the establishment of a T cell-dependent protective immune response.

We next tested whether MK-8722 altered antigen-specific T cell stimulation. As a surrogate of SARS-CoV-2 antigen after infection, we used peptides covering the immunodominant sequence domains of the SARS-CoV-2 Spike (Peptivator peptide pool [Miltenyi]) to stimulate CD14-depleted PBMCs and measure IFNγ^+^ and CD107a^+^ frequencies in both CD4^+^ and CD8^+^ primary T cells. We defined an activation index as the level of activation induced by the peptide pool relative to the corresponding Transact level. For these experiments, cells were collected from healthy individuals after the summer of 2021 when most of them had been immunized at least two times with spike-based SARS-CoV-2 vaccine and consequently mounted a Spike-specific T cell response. Accordingly, the Spike peptide pool stimulated only CD4^+^ T cell response, although in a limited manner (Fig. 5C; activation index: 6.6 ± 0.8 for IFNγ and 1.4 ± 0.3 for CD107a, *t-*test*: P* < 0.01 and *P* < 0.05). As for CD3/CD28 stimulation, MK-8722 stimulation showed a trend toward an increase in all the tested T cell responses, although only CD107a activation index for CD4+ T cells was statistically higher after MK-8722 treatment (i.e., 6.6 ± 0.8 vs 13.3 ± 4.7 for 10 µM MK-8722, ANOVA *P* < 0.01). Stimulation of CD14-depleted PBMCs with a SARS-CoV-2 nucleocapsid peptide pool gave similar results, even though the peptide pool alone failed to activate a T cell response (Fig. S6C). These data suggest that if used as a drug, MK-8722 will likely not prevent the establishment of a T cell-specific response against SARS-CoV-2 *in vivo*.

We finally evaluated whether MK-8722 and SARS-CoV-2-specific CD8^+^ T cells could cooperate to block infection of mucosal epithelial cells. However, Calu-3 cells being HLA-A2 negative, they could not be used to present viral antigen to HLA-A2+ CD8+ T cells. For this reason, instead of Calu-3 cells, we used the epithelial Caco2 cells competent for IFN responses, which expressed HLA-A2 (not shown) and are susceptible to SARS-CoV-2 infection. Therefore, CD8^+^ T cells were obtained from HLA-A2^+^ healthy donors obtained after the summer of 2021. Post-exposure treatment with MK-8722 inhibited infection of Caco2 cells in a dose-dependent manner, as shown in Fig. 5D (by 32% ± 3% and 54% ± 6%, at 2.5 and 5 µM, respectively [ANOVA: *P* < 0.0001 for both conditions], see Fig. S6 for the gating strategy). In the presence of CD8^+^ T cells, infection of Caco2 cells was reduced by 19.8% ± 5% (ANOVA: *P* < 0.01). Moreover, the combination of CD8^+^ T cells with MK-8722 increased the inhibition of infection in a dose-dependent manner (50% ± 3% and 71% ± 4% inhibition in the presence of CD8+ T cells and 2.5 or 5 µM MK-8722, respectively) compared to the inhibition of infection in the sole presence of CD8+ T cells (19.8% ± 5% ANOVA: *P* < 0.0001 both, respectively) and in the presence of MK-8722 only (32% ± 3% and 54% ± 6% in the presence of 2.5 or 5 µM MK-8722, *t*-test *P* < 0.05 and ns for 2.5 and 5 µM, respectively), although no synergy was observed. Altogether, our results confirm that MK-8722 treatment does not impair the SARS-CoV-2-specific CD8^+^ T cell-mediated response against mucosal Caco2 cell infection but rather that both act in combination to reduce cell infection.

## DISCUSSION

To date, antivirals against SARS-CoV-2 infection without significant side effects have yet to be characterized. Here, we provide evidence that the direct pan-AMPK allosteric activator MK-8722 is a strong antiviral drug candidate against SARS-CoV-2 infection, as treatment with MK-8722 in the μM range reduces infection of lung cells without altering the cellular immune response.

Mechanistically, we demonstrate here that AMPK activation by MK-8722 reduces lipid synthesis through the inhibition of ACC by acting on the limiting step of fatty acid production and, consequently, phospholipid synthesis. This inhibition leads to a reduction in the formation of lipid droplets and a significant reduction in the infectivity of viral particles. Lipid synthesis is required to extend the endoplasmic reticulum for the genesis of the viral factories and to generate the viral membrane of SARS-CoV-2, which is an enveloped virus. Changes in the lipid content of viral particles have been shown to impact infectivity (6, 8, 10, 12). AMPK-mediated inhibition of ACC by MK-8722 treatment likely modifies all membrane lipid species in SARS-CoV-2-infected cells (50), in turn affecting viral membrane lipid composition and likely the lower virus infectivity we observed. The modification of the cellular lipid content by MK-8722 treatment might also block the release of pro-inflammatory molecules otherwise induced by infection, such as IL-6 and LTB4 (13, 51). SARS-CoV-2 affects also cholesterol metabolism (8, 52, 53) and patients under statins who contracted SARS-CoV-2 developed better T cell responses and showed a lower grade of pulmonary symptoms (54, 55). AMPK activation also inhibits HMG-CoA reductase, the major enzyme for cholesterol synthesis. MK-8722 treatment might therefore be beneficial for COVID-19 patients through multiple mechanisms (8, 15, 18). The molecular mechanisms underlying the changes in the lipid spectrum and cholesterol content in infected cells following treatment with MK-8722 remain to be understood.

Increased activation of ULK1 and expression of Sequestosome-1/p62 upon MK-8722 treatment reflects a restoration of the autophagy flux, leading to the targeting of viruses to lysosomes for degradation. This mechanism is otherwise blocked by viral replication in an ORF3- and ORF7a-mediated process, which inhibits the HOPS complex-mediated assembly of the SNARE complex required for autolysosome formation (23, 25, 26). Sequestrosome-1/p62 is an autophagy cargo receptor that plays a key role in mediating the formation of autophagosomes and autophagic clearance of intracellular protein aggregates by its binding to LC3b. A modification of p62 expression and LC3b-binding capacity upon SARS-CoV-2 infection has been observed *in vitro* (21, 23, 26). Together, these data suggest that SARS-CoV-2-mediated disruption of p62 expression and/or function impairs the fusion of phagosome with the lysosome (21, 23, 56, 57). The increased p62 expression we observed upon MK-8722 treatment, together with the increased ULK1 activation, will likely result in p62 activation, in turn promoting phagolysosome formation as we reported. However, in contrast to the canonical increase in autophagic flux, in which p62 levels would be expected to decrease, we report an increase in p62 expression levels. Such a phenomenon would depend on p62-mediated KEAP1 degradation following primary AMPK activation and subsequent activation of the p62 transcription factor Nrf2 (42, 58). In addition, as Nrf2 activation is dependent on oxidative stress, its level of activation could be enhanced by the increase in mitochondrial stress during SARS-CoV-2 infection via Nrf2 (59). Convergence of Beclin-Atg14 together with P62 activation could stimulate the selective clearance of viral components in a process called virophagy (60–62). Thus, we propose that AMPK pharmacological activation induces virophagy and is responsible, at least partially, for its antiviral effect. Regrettably, we could not use the commonly used drug chloroquine to show that the restoration of autophagic flux is responsible for the antiviral effect because chloroquine is antiviral against SARS-CoV-2 *in vitro* (63). Nevertheless, as many studies have reported that AMPK activation increases autophagic flux (19, 21, 23, 56, 64) in addition to the evidence provided in our study, we can conclude that normalizing the autophagic flux is antiviral against SARS-CoV-2. However, we cannot rule out that alternative mechanisms such as proteasomal degradation or lysosomal independent pathways could play a role in the observed antiviral activity. Furthermore, P62 also induces proteasomal degradation (65), and its increased expression could contribute to the antiviral activity of MK-8722.

The restoration of autophagy by MK-8722 we report, and thereby degradation of viral components, could improve MHC-I presentation and the cellular cytotoxic response (66), contributing to the therapeutic value of MK-8722 treatment. Furthermore, defective viral particles, which are produced upon MK-8722 treatment, might be detected by innate immune sensors such as MAVS (67), promoting a type-I IFN response and protection from SARS-CoV-2 infection (29). MK-8722 treatment of infected cells also unlocks the IFN-I response, as shown by the increase in *IFNα* and both ISG *Mx1* and *OAS1* mRNA we observed. This IFN-I response is most likely associated with better control of infection and lowers pathology score (27, 28, 30, 44, 68). The induction of IFNβ by poly I:C is only transient, with its mRNA returning even to a level below the basal one (69). Here, treatment with MK-8722 may similarly result in a transient accumulation of *IFNβ* mRNA, though its final level is statistically lower than that observed in the absence of treatment. This faster IFN-I response could enhance the protection of individuals against SARS-CoV-2 since a delay in IFN-I response is associated with disease severity (30, 44) and post-infection treatment of severe COVID with IFNα2b significantly improved patient outcome (45).

Finally, MK-8722 could also enhance the response of SARS-CoV-2-specific CD8^+^ T cells at the site of infection, which is crucial for protection in non-human primates (NHPs) and in humans (47–49). Optimal CD8^+^ T cell responses at the mucosal level can be boosted by IFN-I, which showed vaccine adjuvant-like properties in strategies against other infectious diseases and cancer (70–73). MK-8722 treatment, which stimulates the IFN-I pathway upon infection as shown here, could therefore contribute to improving the CD8^+^ T cell response. Accordingly, *in vitro*, MK-8722 does not antagonize but rather slightly enhances the T cell response, and when combined with CD8^+^ T cells, the resulting treatment increases inhibition of infection compared to either treatment alone. Further studies on the crosstalk between CD8^+^ T cells, phagocytes, and infected epithelial cells will clarify how MK-8722 modulates the CD8^+^ T cell response. The antiviral effect of AMPK activation in primary lung reconstruction and preclinical models such as hamster remains to be tested.

Previous studies have suggested that AMPK activation could inhibit viral infections (35), as viral infection prevents AMPK activity. In the case of SARS-CoV-2 infection, AMPK activity is reduced, and furthermore, autophagy is blocked (19), while inhibition of either AMPK by Compound C or ULK1 by MRT68921 increases viral particle release (19, 74). We show here that the blockade of AMPK activation upon infection can be reversed by MK-8722, the pharmacological allosteric pan-activator of AMPK, which blocks infection at a µM concentration. This result is in agreement with the predicted role of AMPK activity on SARS-CoV-2 infection (75) and with MK-8722 activity on infection by other viruses (76). In line, metformin, a drug approved the FDA since 1994, and the adenosine analog AICAR that activates AMPK indirectly can inhibit replication of SARS-CoV-2 as well as Flaviviruses but at concentrations of 10 and 1 mM, respectively (36, 74). These AMPK-sensitive viruses replicate all in viral factories, disturbing lipid synthesis and escaping autophagy (77, 78). AMPK activation by metformin at high concentration (10 mM) inhibits SARS-CoV-2 replication *in vitro* (74). However, AMPK activation with AICAR, a non-metabolized analog of AMP able to activate AMPK, used at 25 µM is unable to inhibit SARS-CoV-2 infection (19) while, at 1 mM, has been proven to reduce by 10-fold viral (74), suggesting that AMPK needs to reach an activation threshold to inhibit viral replication. Conversely, NUAK2, an AMPK-related kinase, was reported to stimulate viral replication in A549 and Calu3 cells (79). Altogether our results, in agreement with the literature, indicate that AMPK-dependent antiviral activity is restricted to AMPK members only, confirming their distance from AMPK-related kinases such as NUAK2 (80). Despite observational data suggesting an association between metformin use and prevention of severe COVID-19 outcomes, metformin did not significantly lower the risk of hospitalization and mortality due to SARS-CoV-2 infection in recent randomized controlled trials (81). Furthermore, *in vivo*, metformin mainly targets the liver and the gastrointestinal tract (82) and therefore could have limited effect in the upper airway, the SARS-CoV-2 infection site. In contrast, MK-8722, as a systemic drug, may reach these tissues with minimal side effects, as a daily treatment in diabetic NHPs with MK-8722 (10 mg/kg) for a month induced only a limited and reversible cardiac hypertrophy (37). The MK-8722 antiviral treatment we report is efficient *in vitro* not only when applied from the onset of infection but also after infection. Therefore, MK-8722 might be tested as post-exposure prophylaxis against SARS-CoV-2, thereby limiting potential severe outcomes.

In summary, to optimize safe antiviral treatment against SARS-CoV-2, as summarized in the graphical abstract, we propose here to target the energy sensor AMPK using small molecule pan-AMPK allosteric activators (such as MK-8722), rather than targeting the virus itself. The antiviral mechanism of MK-8722 relies on the reversion of three major pathways affected by SARS-CoV-2 infection, namely lipid metabolism, autophagy, and IFN-I response. Therefore, to escape MK-8722 and generate resistant and more pathogenic variants, SARS-CoV-2 will need to acquire a higher number of resistance mutations than a drug targeting a single pathway. Furthermore, by increasing the SARS-CoV-2 CD8^+^ T cell response, MK-8722 treatment would result in a faster response against infection and better outcome. *Coronaviruses*, as well as *Flaviviruses*, replicate within viral factories and/or disturb lipid metabolism, hijack autophagy, and IFN-I response. Therefore, we propose that MK-8722 could act as an antiviral against other *Coronaviruses* and *Flaviviruses*, ensuring a larger available therapeutic arsenal against future emerging virosis.

## Data Availability

All research data associated with the paper can be obtained upon request from M.B.

## SUPPLEMENTAL MATERIAL

Supplemental figures jvi.00394-25-s0001.pdf Figures S1 to S6.

Graphical abstract jvi.00394-25-s0002.docx Graphical representation of study highlights.

ASM does not own the copyrights to Supplemental Material that may be linked to, or accessed through, an article. The authors have granted ASM a non-exclusive, world-wide license to publish the Supplemental Material files. Please contact the corresponding author directly for reuse.
