# Supplementary material for: Direct pharmacological AMPK activation inhibits mucosal SARS-CoV-2 infection by reducing lipid metabolism, restoring autophagy flux and the type I IFN response
Source: J Virol. 2025 Jun 12;99(7):e00394-25. doi: 10.1128/jvi.00394-25 (PMC12282067; doi:10.1128/jvi.00394-25)
Supplement: Supplemental figures — Figures S1 to S6. [file jvi.00394-25-s0001.pdf]

Direct pharmacological AMPK activation inhibits mucosal SARS-CoV-2 infection by reducing lipid metabolism, restoring autophagy flux and the type I IFN response

Cottignies-Calamarte et *al.*

Supplementary Figure Legends

Supplementary Figure S1: Kinetic of AMPK activation and downstream ACC phosphorylation evaluated by western-blot after MK-8722 stimulation of Vero76.

A: AMPK and ACC activation upon MK-8722 treatment. Fold changes of pAMPK/AMPK and pACC/ACC over Non-treated condition were made at each timepoint.

B: **Whole western blot membrane used for β-actin, AMPK and ACC phosphorylation** of Vero76 after different time and dose of MK-8722 treatment. 3 independent lysates were run on the same gel.

Supplementary Figure S2: MK-8722 inhibits SARS-CoV-2 infection.

A: Gating strategies used to analyse infection inhibition by MK-7822 treatment in Vero76 cells in Figure 1B.

B: Cells were treated as indicated in Figure 1B. Frequencies of infection in corresponding experimental sample shown in Figure 1B were measured by SARS-CoV-2 Spike detection in Vero76 cells. Shown are mean ± SEM. n=4 independent experiments.

C: Gating strategies used to analyse infection inhibition by MK-7822 treatment in Calu-3 cells in Figure 1C.

D-E: Cells were treated as indicated in Figure 1B and C. Frequencies of infection in corresponding experimental sample shown in Figure 1B and C were measured by dual detection of Spike (Black) and viral RNA (Red) using Fish-flow in Calu-3 cells infected by Alpha **(D)** or Omicron **(E)** variant, quantified by flow cytometry. Shown are mean ± SEM. n=4 independent experiments.

F: Inhibition of infection by Remdesevir. Calu-3 cells were treated with Remdesivir (0.1 µM or 1 µM) 1h prior and during the 2h of inoculation with SARS-CoV-2 Alpha variant. After virus removal, treatment with Remdesivir at indicated concentration was continued. At 4dpi, infection was quantified by dual detection of Spike (Black) and viral RNA (Red) in Fish-flow quantified by flow cytometry. Shown are mean ± SEM of Spike and vRNA frequency (left) and infection inhibition (right). n=3 independent experiments.

G: Whole western blot membrane used for β-actin and nucleocapsid of both non infected Vero76 and Calu-3 (representative of n=3 independent experiments and corresponding to experiments shown in Fig.1E)

H-I: ACE2 expression and viability were evaluated in Vero76 cells after 24h or in Calu-3 cells after 4days of MK-8722 continuous treatment (1 µM or 5 µM respectively) by flow cytometry. ACE2 expression is expressed as MFI (***G***) and viability as frequency of cells non-stained by the amine-reactive dye Viobility (***H***). n=3 independent experiments.

J: Evaluation of MK-8722 toxicity over time and concentration in Calu-3 cells. n=3 independent experiments.

Shown are mean ± SEM. ANOVA: * p<0.05, ** p<0.01, *** p<0.001, **** p<0.0001.

Supplementary Figure S3: ***Evaluation of metabolism changes as antiviral effect of AMPK-activation by MK-8722 treatment.***

A: Whole membranes used for of both Vero76 and Calu-3 (representative of 2 experiments).

B: Calu-3 cells were left uninfected and treated with MK-8722 (5 µM) or not and analyzed as indicated in Figure 3. Representative fields for Nile Red (Red) and Nucleocapsid N protein immunodetection (Green) imaged by confocal microscopy are shown. Nuclei were stained with DAPI (Blue). Bars: 20 µm

C: Cells were treated and labelled as described in Figure 2. Total intensity of total Nile Red staining per cell was calculated in ImageJ for non-infected non-treated (Ni-NT), infected non-treated (Infected-NT) and Infected MK-8722 treated conditions. Values were normalized to the mean of non-infected non-treated condition (Ni NT). Shown are mean ± SEM. n= 3 independent experiments. ANOVA: p<0.05.

Supplementary Figure S4: MK-8722 restores lysosomal parameters in Calu-3 cells.

A: Whole membranes used for of both Vero76 and Calu-3 (representative of 2 experiments).

B: Cells were left uninfected but treated with MK-8722 (5 µM) or not and analysed as indicated in Figure 4. Representative fields for LAMP1 (Red) and Nucleocapsid N protein (Green) immunodetection imaged by confocal microscopy are shown. Nuclei were stained with DAPI (Blue). Bars: 20 µm

C: Cells were treated and labelled as described in Figure 4. Total intensity of total LAMP1 staining per cell was calculated in ImageJ for non-infected non-treated condition (Ni-NT), infected non-treated (Infected-NT) and treated with 5 µM MK-8722 (Infected-MK-8722). Values were normalized to corresponding non-infected non-treated condition. n≥3 independent experiments. Shown are mean ± SEM. ANOVA: ** p<0.01 and *** p<0.001.

Supplementary Figure S5: Evaluation of nucleocapsid-specific T cell response in SARS-CoV-2 vaccinated individuals upon MK-8722 treatment ex vivo.

A: PBMCs from healthy donors were cultivated for 6h indicated concentration of MK-8722 or corresponding DMSO serving as carrier. CD4 and CD8 T cells activation (CD107a and IFNg expression) was monitored by flow cytometry.

B: Gating strategy to investigate $CD4^+$ and $CD8^+$ T cell responses stimulation after activation with anti-CD3/anti-CD28 antibodies (Transact) with or without MK-8722 treatment as analysed in Figure 5A.

C: CD14-depleted PBMCs corresponding to donors used in Figure 5 were stimulated as described in Figure 5B but with T cell Nucleocapsid peptides (Peptivator, Miltenyi), and monitored by flow cytometry for IFNγ and CD107a expression. Activation levels are presented as activation index as described in the methods section. $n \geq 3$ independent donors.

Supplementary Figure 6: Gating strategy for combined MK-8722 treatment and $CD8^+$ T cell antiviral activity in coculture.

Gating strategy to quantify the inhibition of Caco2 cells infection in the presence of MK-8722 and/or $CD8^+$ T cells from vaccinated individuals shown in Figure 5C. Infection was evaluated within Caco2 cells corresponding to the $CD3^-CD8^-$ population. The low number of $CD8^+$ T cells (around 150) prevented us from investigating their activation level.

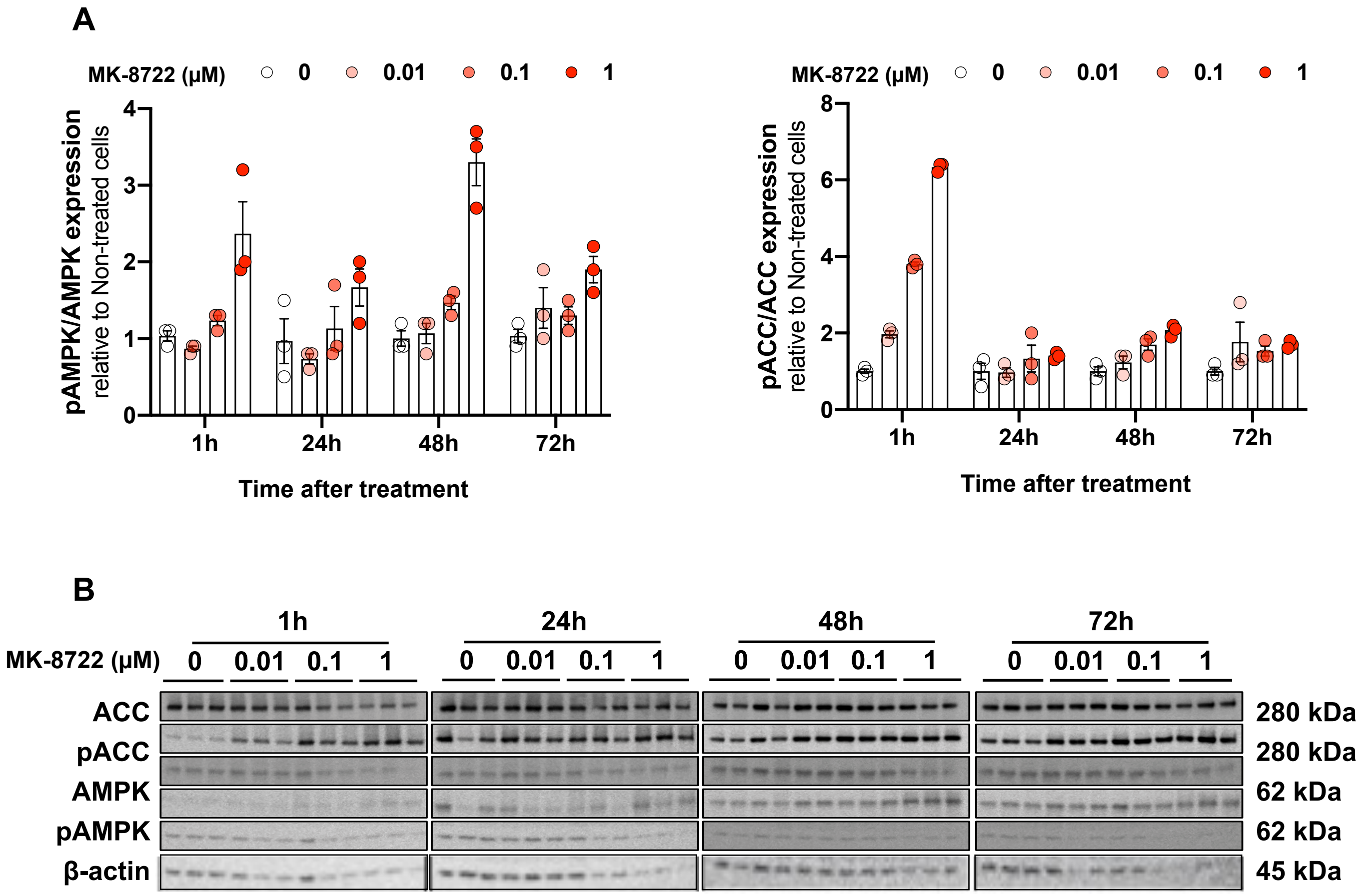
A
MK-8722 (μM)
0
0.01
0.1
1
pAMPK/AMPK expression
relative to Non-treated cells
1h
24h
48h
72h
Time after treatment
pACC/ACC expression
relative to Non-treated cells
B
MK-8722 (μM)
ACC
pACC
AMPK
pAMPK
β-actin
280 kDa
280 kDa
62 kDa
62 kDa
45 kDa

Figure S1

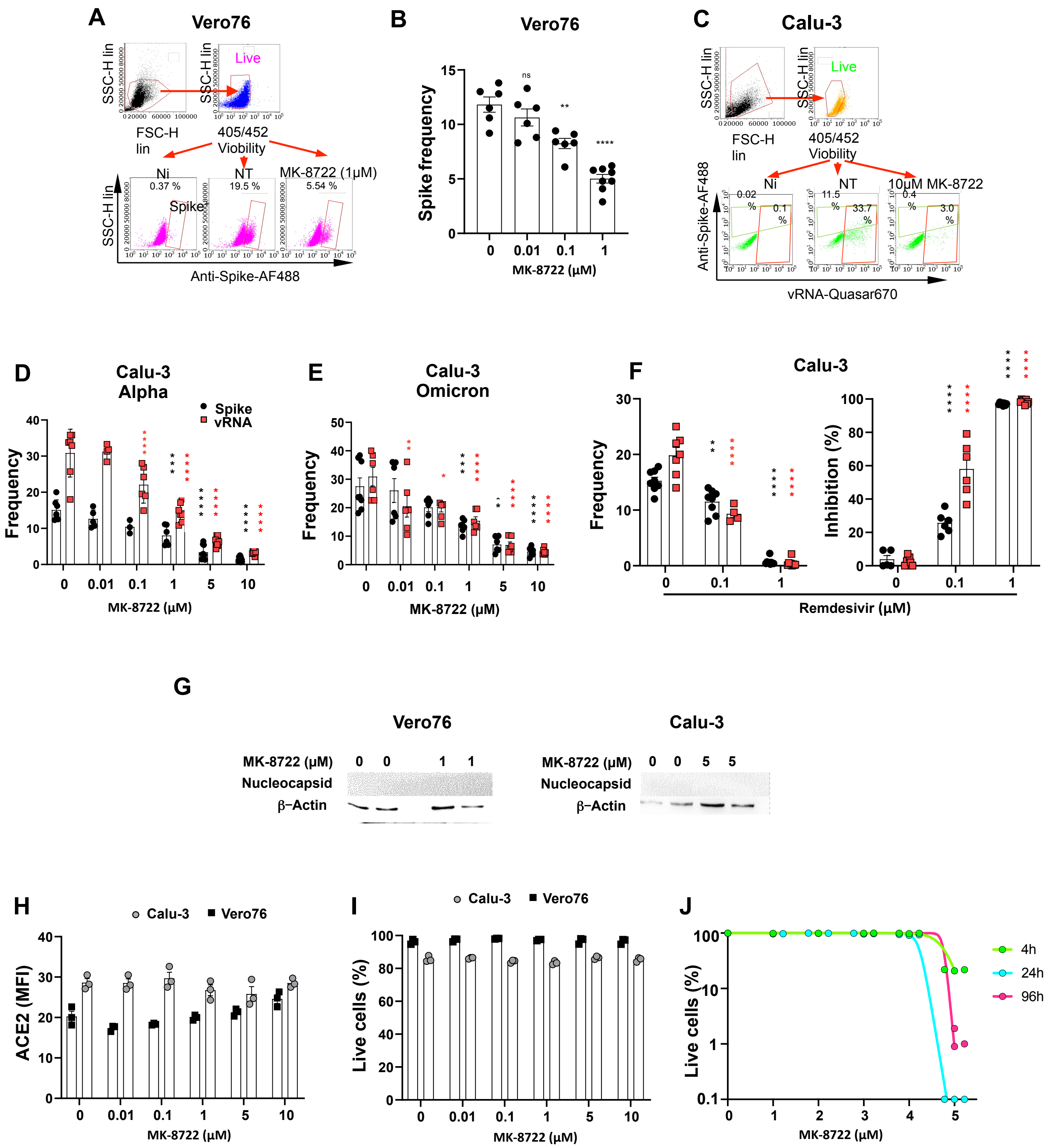

A
Vero76
SSC-H lin
FSC-H lin
Live
405/452 Viobility
Ni
NT
MK-8722 (1μM)
0.37 %
19.5 %
5.54 %
Spike+
Anti-Spike-AF488
B
Vero76
Spike frequency
MK-8722 (μM)
C
Calu-3
Live
405/452 Viobility
Ni
NT
10μM MK-8722
Anti-Spike-AF488
vRNA-Quasar670
D
Calu-3 Alpha
Spike
vRNA
Frequency
MK-8722 (μM)
E
Calu-3 Omicron
Frequency
MK-8722 (μM)
F
Calu-3
Frequency
Inhibition (%)
Remdesivir (μM)
G
Vero76
Calu-3
MK-8722 (μM)
Nucleocapsid
β−Actin
H
Calu-3
Vero76
ACE2 (MFI)
MK-8722 (μM)
I
Calu-3
Vero76
Live cells (%)
MK-8722 (μM)
J
Live cells (%)
4h
24h
96h
MK-8722 (μM)

Figure S2

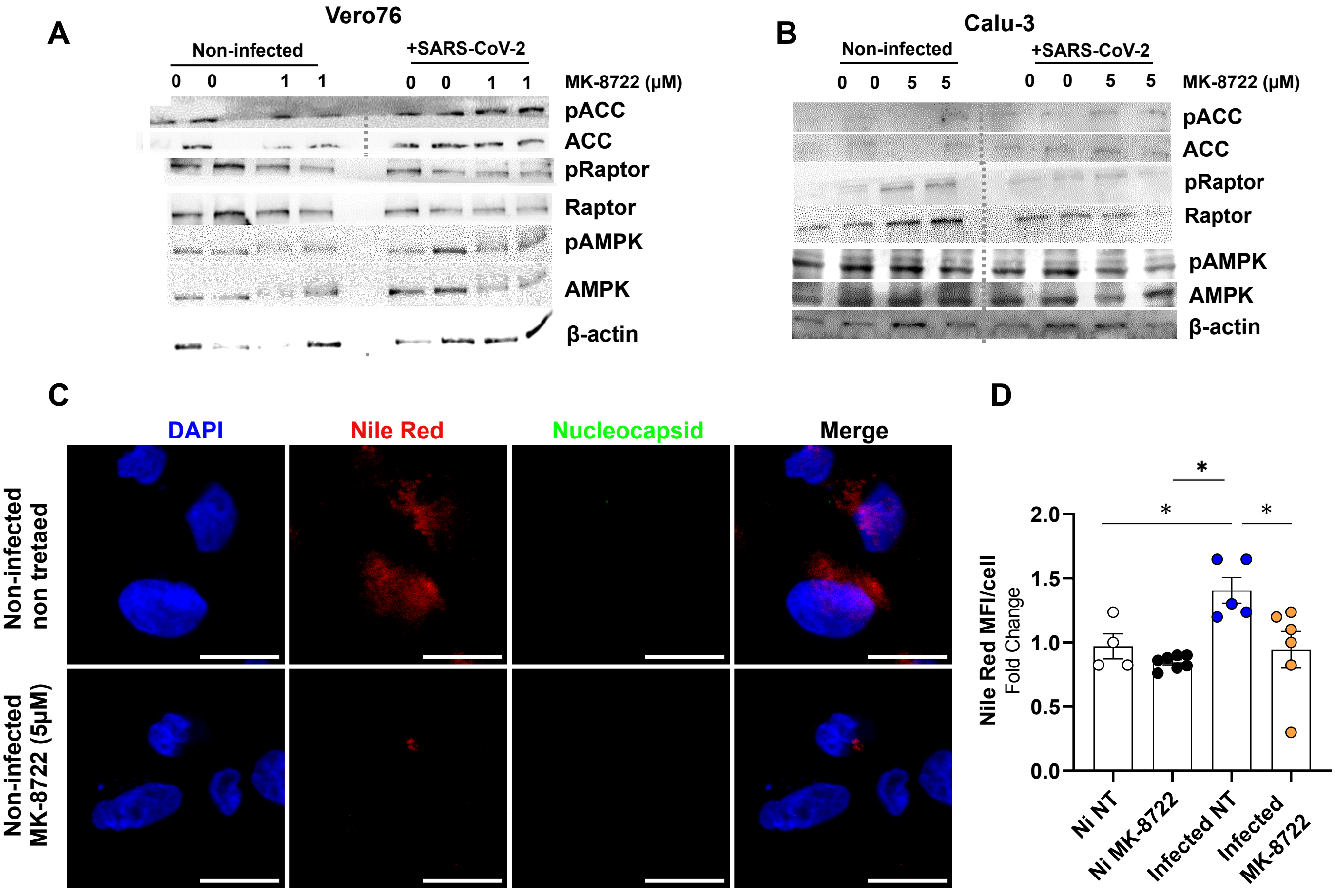
A
Vero76
Non-infected
+SARS-CoV-2
0 0 1 1 0 0 1 1
MK-8722 (μM)
pACC
ACC
pRaptor
Raptor
pAMPK
AMPK
β-actin
B
Calu-3
Non-infected
+SARS-CoV-2
0 0 5 5 0 0 5 5
MK-8722 (μM)
pACC
ACC
pRaptor
Raptor
pAMPK
AMPK
β-actin
C
DAPI
Nile Red
Nucleocapsid
Merge
Non-infected non tretaed
Non-infected MK-8722 (5μM)
D
Nile Red MFI/cell Fold Change
2.0
1.5
1.0
0.5
0.0
*
*
*
Ni NT
Ni MK-8722
Infected NT
Infected MK-8722

Figure S3

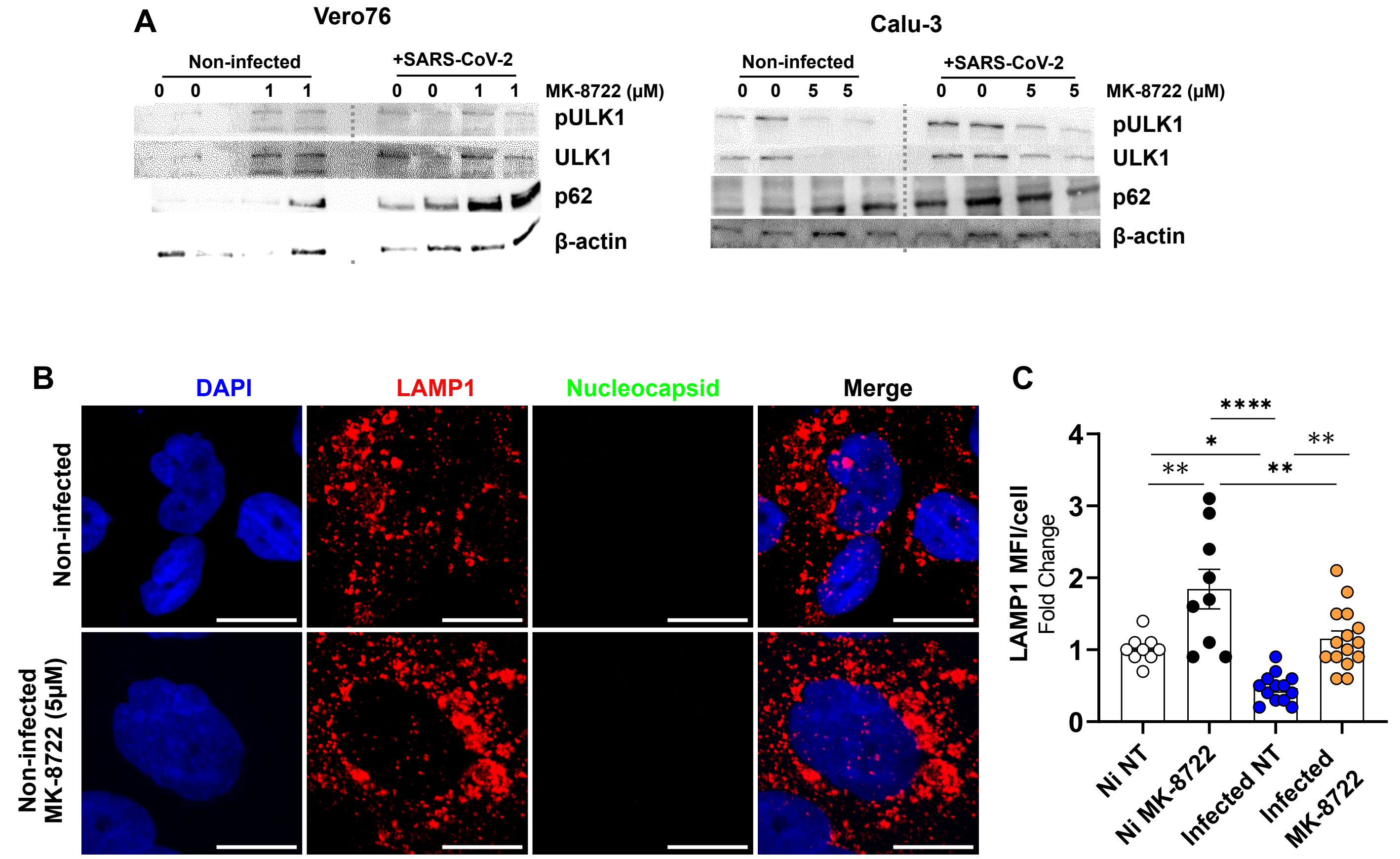
A
Vero76
Non-infected
+SARS-CoV-2
0 0 1 1 0 0 1 1
MK-8722 (μM)
pULK1
ULK1
p62
β-actin
Calu-3
Non-infected
+SARS-CoV-2
0 0 5 5 0 0 5 5
MK-8722 (μM)
pULK1
ULK1
p62
β-actin
B
DAPI
LAMP1
Nucleocapsid
Merge
Non-infected
Non-infected MK-8722 (5μM)
C
LAMP1 MFI/cell
Fold Change
0
1
2
3
4
****
*
**
**
**
Ni NT
Ni MK-8722
Infected NT
Infected MK-8722

Figure S4

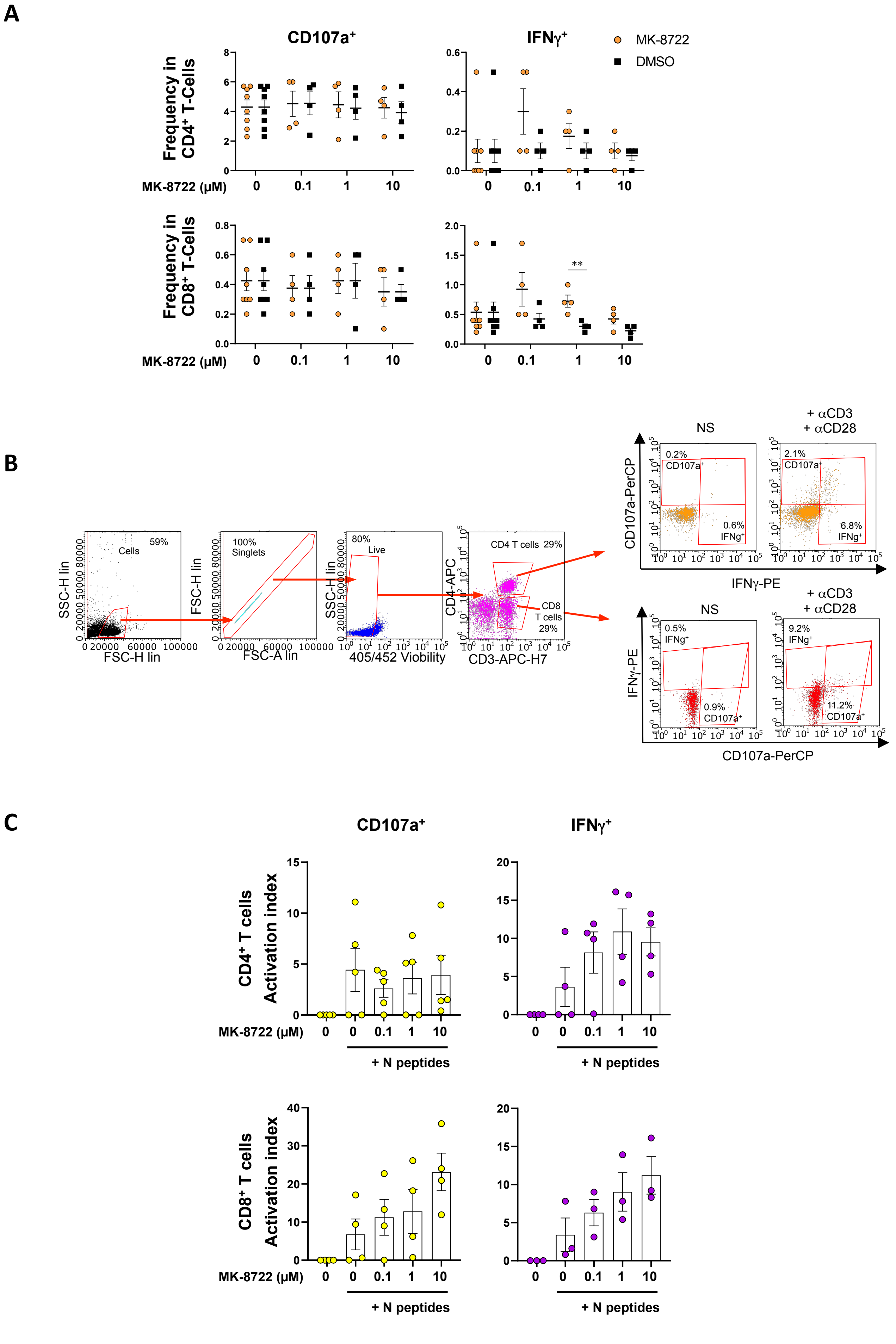
A
CD107a⁺
IFNγ⁺
MK-8722
DMSO
Frequency in CD4⁺ T-Cells
Frequency in CD8⁺ T-Cells
MK-8722 (µM)
0
0.1
1
10
**
B
Cells 59%
100% Singlets
80% Live
CD4 T cells 29%
CD8 T cells 29%
SSC-H lin
FSC-H lin
FSC-A lin
405/452 Viobility
CD4-APC
CD3-APC-H7
NS
+ αCD3 + αCD28
0.2% CD107a⁺
0.6% IFNg⁺
2.1% CD107a⁺
6.8% IFNg⁺
CD107a-PerCP
IFNγ-PE
0.5% IFNg⁺
0.9% CD107a⁺
9.2% IFNg⁺
11.2% CD107a⁺
C
CD107a⁺
IFNγ⁺
CD4⁺ T cells Activation index
CD8⁺ T cells Activation index
MK-8722 (µM)
+ N peptides

Figure S5

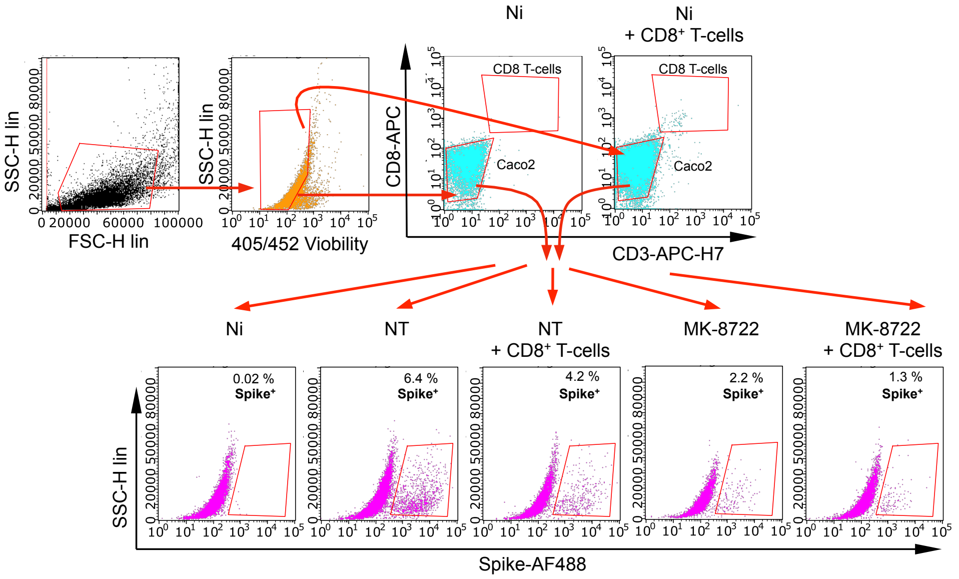

SSC-H lin
FSC-H lin
405/452 Viobility
Ni
Ni
+ CD8⁺ T-cells
CD8-APC
CD8 T-cells
Caco2
CD3-APC-H7
Ni
NT
NT
+ CD8⁺ T-cells
MK-8722
MK-8722
+ CD8⁺ T-cells
0.02 %
Spike⁺
6.4 %
Spike⁺
4.2 %
Spike⁺
2.2 %
Spike⁺
1.3 %
Spike⁺
Spike-AF488

Figure S6
