# Supplementary material for: Direct pharmacological AMPK activation inhibits mucosal SARS-CoV-2 infection by reducing lipid metabolism, restoring autophagy flux and the type I IFN response
Source: J Virol. 2025 Jun 12;99(7):e00394-25. doi: 10.1128/jvi.00394-25 (PMC12282067; doi:10.1128/jvi.00394-25)
Supplement: Graphical abstract — Graphical representation of study highlights. [file jvi.00394-25-s0002.docx]

**Graphical abstract**

## Highlights

- **MK-8722 exerts post-exposure antiviral activity**
- **MK-8722 induces a decrease in cellular lipid content**
- **MK-8722 promotes an increase in the autophagic flux of viral components**
- **MK-8722 promotes the restoration of the IFN-I activity**

**MK-8722 antiviral activity is compatible with virus-specific T cell responses**
